# Investigation and Distinction of Energy Metabolism in Proliferating Hepatocytes and Hepatocellular Carcinoma Cells

**DOI:** 10.3390/cells14161254

**Published:** 2025-08-14

**Authors:** Julia Nerusch, Gerda Schicht, Natalie Herzog, Jan-Heiner Küpper, Daniel Seehofer, Georg Damm

**Affiliations:** 1 Department of Hepatobiliary Surgery and Visceral Transplantation, University Hospital, Leipzig University, 04103 Leipzig, Germany; 2 Saxonian Incubator for Clinical Translation (SIKT), Leipzig University, 04103 Leipzig, Germany; 3 Faculty of Science, Brandenburg University of Technology Cottbus-Senftenberg, 01968 Senftenberg, Germany

**Keywords:** hepatocellular carcinoma, HepaFH3, metabolic reprogramming, glycolysis, ketone body metabolism, liver regeneration, tumor markers, *HIF1A*, *c-MYC*, *BDH1*

## Abstract

Metabolic rewiring is a hallmark of both hepatic regeneration and malignant transformation, complicating the identification of cancer-specific traits. This study aimed to distinguish the metabolic profiles of proliferating hepatocytes and hepatocellular carcinoma (HCC) cells through integrated analyses of mRNA and protein expression, along with functional characterization. We compared non-malignant Upcyte^®^ hepatocytes (HepaFH3) cultured under proliferative and confluent conditions with primary human hepatocytes, primary human hepatoma cells, and hepatoma cell lines. Proliferating HepaFH3 cells exhibited features of metabolic reprogramming, including elevated glycolysis, increased *HIF1A* expression, and ketone body accumulation, while maintaining low c-MYC expression and reduced *BDH1* levels, distinguishing them from malignant models. In contrast, HCC cells showed upregulation of HK2, c-MYC, and *BDH1*, reflecting a shift toward aggressive glycolytic and ketolytic metabolism. Functional assays supported the transcript and protein expression data, demonstrating increased glucose uptake, elevated lactate secretion, and reduced glycogen storage in both proliferating and malignant cells. These findings reveal that cancer-like metabolic changes also occur during hepatic regeneration, limiting the diagnostic utility of individual metabolic markers. HepaFH3 cells thus provide a physiologically relevant in vitro model to study regeneration-associated metabolic adaptation and may offer insights that contribute to distinguishing regenerative from malignant processes. Our findings highlight the potential of integrated metabolic profiling in differentiating proliferation from tumorigenesis.

## 1. Introduction

The liver possesses a remarkable regenerative capacity, enabling it to restore lost tissue following injuries or major surgical procedures such as partial hepatectomy [1,2]. This regenerative process involves extensive cellular proliferation and metabolic reprogramming. Mature hepatocytes display different metabolic profiles compared to regenerating hepatocytes, reflecting these adaptations [3]. Interestingly, many of the underlying mechanisms driving liver regeneration overlap with those activated in cancer. Cancer does not invent new biological programs but often hijacks existing ones, such as proliferation, survival, and metabolism, that are inherent to normal tissue responses [4,5]. As a result, proliferating hepatocytes during regeneration and hepatocellular carcinoma (HCC) cells may share similar metabolic traits. However, this biological overlap presents a challenge: it becomes difficult to determine which metabolic changes are truly cancer-specific and which are simply associated with increased proliferation. Clarifying the differences is crucial for identifying reliable biomarkers of malignancy and developing therapies that selectively target tumor metabolism without impairing the liver’s regenerative capacity.

Primary liver cancer ranks among the six most prevalent cancers worldwide, being the third leading cause of cancer-related death [6]. HCC accounts for approximately 80% of all primary liver cancer cases, while 15% are classified as cholangiocarcinoma [7]. The etiology of HCC is multifactorial, influenced by chronic infections with hepatitis B and C viruses, alcohol consumption, exposure to aflatoxins, and metabolic dysfunction-associated steatotic liver disease (MASLD) [8,9,10].

A hallmark of HCC pathophysiology is the reprogramming of cellular metabolism [5,11]. One significant characteristic is the Warburg effect, which involves a shift to aerobic glycolysis, marked by increased glucose uptake and lactate production, even in the presence of oxygen [12]. This metabolic adaptation supports rapid cell proliferation, resistance to apoptosis and tumor progression. However, widely used HCC models such as hepatoma cell lines (HCLs) HepG2 and Huh7, as well as primary human hepatoma cells (PHCs), have been shown to only partially reflect the metabolic characteristics of actual HCC. Interestingly, primary human hepatocytes (PHHs) isolated from HCC patients display metabolic features similar to those of PHCs, suggesting that these alterations may occur already in healthy tissue at the early stages of tumor development [13].

In contrast to cancer cells, the metabolism of quiescent PHHs is primarily regulated to support the liver’s specific synthetic and metabolic functions. However, during liver regeneration, hepatocytes enter a proliferative state that demands substantial energy. To meet this requirement, a metabolic reprogramming occurs, including shifts in enzyme expression and activity [14,15]. Proliferating cells are thought to adopt a Warburg-like metabolic profile, relying predominantly on glycolysis for energy production, lactate secretion, and mitochondrial activity—even under normoxic conditions [4].

HepaFH3 cells have been proposed as a suitable in vitro model for studying proliferating human hepatocytes. These cells derived from PHHs have been transduced with Upcyte^®^ proliferation genes using a lentiviral vector system [16,17]. They have been characterized in applications related to liver function, toxicology, and drug metabolism research [18]. Notably, HepaFH3 cells can be cultured in two distinct states: a confluent state, which displays more differentiated and quiescent features, and a proliferating state, which exhibits metabolic adaptations similar to those seen in HCLs. The proliferating state is thus considered a non-malignant proliferating cell model with altered metabolism, while the confluent state more closely mimics the metabolic characteristics of non-proliferating PHHs [19].

In healthy hepatocytes, glucose metabolism is primarily regulated by glucokinase (GCK), which catalyzes the initial step of glycolysis in response to elevated blood glucose levels, thereby enabling glycogen synthesis [20]. However, in HCC, an isoenzyme shift occurs: GCK is downregulated and replaced by hexokinase 2 (HK2), which has a higher affinity for glucose and operates independently of insulin regulation [21,22]. This enzymatic shift enhances glucose turnover and supports the increased energy demands of tumor growth [23]. Elevated HK2 expression is associated with poor prognosis in HCC patients, underscoring its critical role in tumor progression [24]. In parallel, a shift in glucose transport mechanisms occurs, with cancer cells favoring glucose transporters (GLUT1) over the hepatocyte-specific GLUT2 [25,26], and the pyruvate kinase M2 isoform (PKM2) is favored over pyruvate kinase liver (PKL), further promoting the Warburg effect by upregulating hypoxia-inducible factor 1 (HIF1) and MYC proto-oncogene (c-MYC) expression [27,28,29,30].

This metabolic rewiring observed in HCC is orchestrated by key transcription factors and oncogenes. The proto-oncogene c-MYC plays a central role in promoting the Warburg effect by upregulating the expression of GLUT1 and glycolytic enzymes such as HK2, PKM2, and lactate dehydrogenase (LDHA) [31,32]. These changes support rapid energy production and biosynthesis essential for continuous proliferation. Additionally, c-MYC contributes to the creation of a hypoxic microenvironment by enhancing the expression of HIF1A, which further increases the glycolytic flux by inducing glycolysis-associated genes like *GLUT1*, *LDHA*, *PKM2*, and *HK2* [30,33,34,35]. Together, these factors reinforce a metabolic state that favors tumor growth and enhances resistance to cellular stress [35].

Under nutrition-limited conditions, such as fasting or tumor-associated hypoxia, the liver initiates ketogenesis to provide alternative energy sources. While normal hepatocytes do not metabolize ketone bodies themselves, HCC cells have been shown to exploit this pathway by utilizing ketone bodies like ß-hydroxybutyrate (β-HB) as an energy source [36]. This adaptation supports tumor cell survival during nutrient deprivation and has been associated with suppression of autophagy, thereby promoting tumor progression and metastasis [36,37]. These findings highlight that both glycolytic and ketone metabolic pathways are altered in HCC to sustain malignancy, complicating the differentiation of cancer-specific adaptations from normal regenerative processes.

Further research is required to clarify which metabolic pathways are specifically altered during malignant transformation, as opposed to those merely associated with regeneration. This distinction is critical for identifying metabolic biomarkers that are truly cancer specific. In this study, we aimed to define the metabolic signature of proliferating hepatocytes and to distinguish it from HCC. To this end, we used Upcyte^®^ hepatocytes (HepaFH3), a lentiviral transfected cell model that mimics proliferative human hepatocytes, and compared them to established hepatoma cell lines (HepG2 and Huh7) as well as to PHCs isolated from HCC patients. Using transcript-, protein-, and metabolic profiling, we assessed hepatic differentiation markers, tumor-associated genes, and their key metabolic enzymes. Our findings suggest that HepaFH3 cells exhibit a distinct metabolic phenotype, differentiating them from both malignant and non-malignant hepatocytes, making them a promising model for investigating regeneration-associated metabolic reprogramming in the liver.

## 2. Materials and Methods

### 2.1. Isolation of Primary Liver Cells and Cell Culture

Isolated primary human cells (PHHs, PHCs) and cell lines (HepG2, Huh7, HepaFH3) were either freshly isolated from liver tissue or obtained as cryopreserved cells. The tissues required for the isolation of PHHs and PHCs were provided by the Department of Hepatobiliary Surgery and Visceral Transplantation at the University Hospital of Leipzig, Germany, with patient informed consent (Table 1). The study was conducted in accordance with the Declaration of Helsinki and approved by the Ethics Committee of the Medical Faculty of Leipzig University under the following protocols: 422/21-ek (SMART-NAFLD study), 322/17-ek (biobanking and use of primary human liver tissues), and 178/16-lk (IMOMESIC project). All liver tissue samples were obtained with written informed consent from donors. Additional details are provided in the Institutional Review Board Statement.

#### 2.1.1. Isolation and Culturing of Primary Human Hepatocytes

Liver tissue excision was conducted in the operating room under sterile conditions. Tissue was immediately transported in cold PHH culture medium (500 mL William’s E medium with GlutaMAX, supplemented with 10% FBS Superior (Merck KgaA, Darmstadt, Germany), 1 µg/mL dexamethasone (Jenapharm GmbH + Co., KG, Jena, Germany), 32 U/L insulin (Eli Lilly and Company, Indianapolis, IN, USA), 1.5% HEPES, 1% MEM NEAA 100×, 1% sodium pyruvate, and 100 U/100 µM penicillin/streptomycin (all provided by Thermo Fisher Scientific, Waltham, MA, USA)) with an average cold ischemia time of 20 min. PHHs were isolated from tumor-free liver resections by a two-step EGTA-collagenase perfusion procedure as previously described [39,40,41]. Isolated cells were resuspended in PHH culture medium. Viability and cell count were assessed using a Neubauer chamber (Th. Geyer GmbH & Co., KG, Renningen, Germany) with trypan blue (Sigma–Aldrich, St. Louis, MO, USA) exclusion, and validated via the Muse^®^ cell counting device (Merck Millipore, Merck KgaA) using the manufacturer’s protocol. PHHs were plated at a density of 200,000 cells/cm^2^ on collagen I-coated plates (0.02 mg/mL) and cultured for 16 h at 37 °C, 5% CO_2_. The medium was then replaced with serum-free starvation medium (PHH culture medium without FBS, dexamethasone, or insulin) for 4 h. Supernatants and cells were then harvested.

#### 2.1.2. Isolation of Primary Human Hepatoma Cells

PHCs were isolated from resected liver tumor tissue macro- and microscopically confirmed as HCC. Isolation was performed in accordance with established methods [13]. In brief, tumor tissue was excised, rinsed, and enzymatically digested using collagenase (Roche, Basel, Switzerland) and DNase (Merck KgaA). The isolated cells were resuspended in PHC culture medium (DMEM supplemented with 10% FBS superior, 100 U/100 μM penicillin/streptomycin, 1% MEM NEAA 100×, 1.5% HEPES, and 1% sodium pyruvate). The cell suspension was purified by density gradient centrifugation using a Percoll^®^ solution with a density of 1.03 g/mL and 1.07 g/mL (Sigma–Aldrich). The isolated cells were centrifuged, and the resulting pellets were immediately frozen and stored at −80 °C for further analysis.

#### 2.1.3. Generation of Upcyte^®^ Hepatocytes

Upcyte^®^ hepatocytes are PHHs genetically modified using Medicyte’s proprietary Upcyte^®^ technology [17]. To induce proliferation, cells were transfected with a lentiviral vector system carrying proliferation-inducing genes, as previously described [16]. The HepaFH3 clone originated from a 59-year-old female patient diagnosed with cancer of unknown primary (CUP) and class I obesity. The patient had no known secondary diagnoses or history of chemotherapy, had serum without pathological findings, and was not on long-term medication. The cryopreserved cell clone was provided by the working group of Küpper from the Brandenburg University of Technology in Cottbus-Senftenberg, Germany [18].

#### 2.1.4. Culturing of Cell Lines

Cryopreserved cells were thawed and cultured in PHH- (HepaFH3) or HepG2-/Huh7-culture medium (containing DMEM supplemented with 10% FBS superior, 100 U/100 μM penicillin/streptomycin, and 1% L-glutamine 200 mM). HepaFH3 cells were seeded at 5000 cells/cm^2^ on collagen I-coated plates, while the HCLs (HepG2, Huh7) were seeded at 10,000 cells/cm^2^. The medium was replaced three times per week until a sufficient number of cells were obtained. The cells were detached using trypsin 0.25%/EDTA 0.02% (PAN-Biotech GmbH, Aidenbach, Germany) for 5 min at 37 °C, 5% CO_2_. The cells were centrifuged at 300× *g* for 5 min, resuspended, and counted as previously described. Subsequently, HCLs were plated at a density of 200,000 cells/cm^2^ while HepaFH3 cells were plated at a density of 40,000/cm^2^ and incubated in the respective culture medium for 16 h at 37 °C, 5% CO_2_. To achieve the confluent state in HepaFH3, cells were cultured for an additional seven days. Thereafter, the medium was replaced with a serum-free starvation medium (HCLs without FBS and L-glutamine) for 4 h at 37 °C, 5% CO_2_. The cells were then harvested, and the supernatants were collected for further analysis.

### 2.2. RNA Isolation and RT-qPCR

RNA was extracted from cultured cells or cell pellets using the RNeasy Kit (QIAGEN, Hilden, Germany) for cell lines and RNA-Solv^®^ (VWR International GmbH, Radnor, PA, USA) for primary cells, according to the manufacturer’s instructions. The concentration, purity, and integrity of the RNA were evaluated using a NanoDrop 2000 spectrophotometer (Thermo Fisher Scientific). The extracted RNA was frozen and stored at −80 °C. Reverse transcription was performed using the QuantiTect^®^ Reverse Transcription Kit (QIAGEN) according to the manufacturer’s protocol. The incubation steps were conducted using a thermocycler (ProFlex™, Applied Biosystems, Thermo Fisher Scientific). Primers for the genes listed in Table S1 were purchased from QIAGEN, while gene-specific intron-spanning primers were designed for the genes listed in Table 2 and obtained from biomers.net GmbH (Ulm, Germany). RT-qPCR was performed with the QuantiNova^®^ SYBR^®^ Green PCR Kit (QIAGEN) in accordance with the manufacturer’s instructions. The samples were analyzed in duplicate with 25 ng cDNA using the thermocycler 7500 Real-Time PCR and the 7500 Software v2.0.6 (Applied Biosystems, Thermo Fisher Scientific). The reference genes glucuronidase β (*GUSB*), glyceraldehyde-3-phosphate dehydrogenase (*GAPDH*), and 18S ribosomal RNA (*RRN18S*) were used for normalization. The cycling conditions included an initial heat activation (120 s at 95 °C) followed by 40 cycles of 10 s denaturation at 95 °C and 30 s annealing/extension at 60 °C. The RT-qPCR methodology adhered to the MIQE guidelines [42], and relative gene expression was normalized according to the method proposed by Taylor et al. [43].

### 2.3. Functional Assays

#### 2.3.1. Lactate Assay

Lactate is produced from pyruvate and metabolized during anaerobic glycolysis, a process that occurs in the absence of oxygen. According to the Warburg effect, tumor cells preferentially utilize anaerobic glycolysis for energy production even under normoxic conditions, resulting in increased lactate production compared to normal cells [12]. Lactate levels in supernatants were quantified using a lactate enzymatic UV assay (DIALAB GmbH, Wiener Neudorf, Austria) following the manufacturer’s instructions. Calibration was performed with Diacal Auto multi-calibration serum, and Diacon N multi-control serum normal served as the positive control (both from DIALAB GmbH). In brief, 200 µL of the reaction reagent was mixed with 2 µL of each sample, standard, and positive control, followed by incubation at 37 °C for 5 min. Absorbance was measured at 340 nm using a microplate reader (Synergy H1, BioTek, Winooski, VT, USA).

#### 2.3.2. Glucose Assay

To support proliferation and growth, tumor cells reprogram their glucose metabolism [44]. Glucose concentrations in supernatants were measured using the glucose GOD-PAP-assay according to the manufacturer’s protocol. Diacal Auto multi-calibration serum was used for calibration, and Diacon N multi-control serum normal served as a positive control (both obtained from DIALAB GmbH). In brief, 200 µL of the reagent reaction mix was added to 2 µL of each sample, standard, and positive control, followed by a 10 min incubation at 37 °C. Absorbance was measured at 550 nm using a microplate reader. Glucose consumption or production was calculated by subtracting the glucose concentration measured in each sample from that of the original cell culture medium, as specified by the manufacturer.

#### 2.3.3. Glycogen Assay

The liver plays a central role in glycogen storage and the regulation of blood glucose homeostasis [45]. In cancer cells, glycogen metabolism is reprogrammed to support tumor progression [46]. Glycogen content was determined using amyloglucosidase digestion followed by glucose quantification, according to established protocols [47,48,49]. After cultivation, cells were detached, frozen, and stored at −80 °C until analysis. A standard curve using oyster glycogen (250–2000 µg/mL) and an amyloglucosidase control (both from Sigma–Aldrich) were prepared and treated identically as the samples. Frozen cell pellets were thawed and lysed by sonication for 10 min (SONOREX Super RK 100, BANDELIN electronic GmbH & Co. KG, Berlin, Germany), and the protein content was measured for normalization. To extract residual glucose, 7% perchloric acid (Sigma–Aldrich) was added, mixed for 5 s, and neutralized with 0.5 M sodium hydroxide (Carl Roth, Karlsruhe, Germany). Samples were heated at 100 °C for 10 min. The solution was then treated with 2 M acetate buffer (pH 4.5, containing 4 mg/mL amyloglucosidase, 2 M potassium acetate, and 2 M acetate (all from Carl Roth)), followed by a 2 h incubation at 55 °C. Glucose was then quantified as described previously.

#### 2.3.4. Ketone Body Assay

In conditions of nutrient deprivation, hepatocellular carcinoma cells may utilize ketone bodies as an alternative energy source [36]. β-HB was quantified using the β-Hydroxybutyrate Colorimetric Assay Kit (Cayman Chemical, Ann Arbor, MI, USA) following the manufacturer’s instructions. Cultured cells were scraped, suspended in cold assay buffer, sonicated, and stored at −80 °C. Standards (0–0.5 mM β-HB) were processed identically to samples. After the addition of the developer solution (enzyme mix + WST-1), samples were incubated at room temperature (RT) for 30 min. Absorbance was measured at 445 nm using a microplate reader.

#### 2.3.5. Albumin ELISA

Albumin, a distinctive marker of hepatocyte function, is synthesized and secreted by the liver [50]. Albumin levels were measured using a sandwich ELISA (Biomol GmbH, Hamburg, Germany). ELISA-compatible plates (Greiner Bio-One GmbH, Frickenhausen, Germany) were coated with 50 mM KHCO_3_ (pH 9.6, Carl) containing a 1:100 dilution of goat anti-human albumin antibody (Biomol GmbH) and incubated for 1 h at RT. Plates were washed five times with washing buffer (pH 8.0, 50 mM Trizma Base, 0.05% Tween 20 (both provided by Sigma–Aldrich), 140 mM NaCl (Carl Roth)) and blocked with blocking buffer (pH 8.0, 50 mM Trizma Base, 1% bovine serum albumin (BSA) (by Sigma–Aldrich), 140 mM NaCl) for 30 min at RT. Samples were then added and incubated for 1 h at RT, followed by washing and adding the detection antibody (goat anti-human albumin, HRP-conjugated detection antibody, Biomol GmbH, diluted (1:40,000) in diluent buffer (pH 8.0, 50 mM Trizma Base, 0.05% Tween 20, 1% BSA, 140 mM NaCl)). After incubating for 1 h at RT in the dark and washing five times, TMB (3, 3′, 5, 5; -tetramethylbenzidine) chromogen solution (TMB one component HRP microwell substrate, Biomol GmbH) was added and incubated for 15 min at RT in the absence of light. Subsequently, the reaction was terminated with a stop solution (1% sulfuric acid, Carl Roth), and a color change from blue to yellow was observed. A human reference serum (Biomol GmbH) diluted to 6.125–400 ng/mL served as the standard. Absorbance was measured at 450 nm using a microplate reader within an optical density (OD) reference range of 1.8–2.2. The albumin concentration was calculated using a standard curve with logistic regression with a four-parameter fit and a log axis (4PL).

#### 2.3.6. Protein Quantification

A bicinchoninic acid (BCA) assay was used to quantify and normalize protein concentrations. Cells were lysed with either BCA buffer (DPBS with 0.1% SDS, 0.5% Triton X-100, and 50 mM Trizma HCl (all provided by Sigma–Aldrich)) for general normalization or RIPA buffer (Tris 50 mM (pH 7.4), 150 mM NaCl (both provided by Carl Roth), 1 mM EDTA (pH 8.8), 0.5% sodium deoxycholate, 0.5 mM Na_3_VO_4_ (all provided by Sigma–Aldrich, St. Louis, MO, USA), 2.5 mM NaF (Honeywell Specialty Chemicals Seelze GmbH, Seelze, Germany) in ddH_2_O mixed with proteinase inhibitors 0.1% aprotinin, 0.1% 4-(2-aminoethyl)benzolsulfonylfluoride (AEBSF) and 1% Nonidet P-40 (all provided by Sigma–Aldrich)) for Western blot analysis. Following lysis, cells were scraped and frozen overnight at −20 °C. Lysates were thawed, centrifuged at 20,000× *g* for 20 min at 4 °C, ultrasonicated for 30 s, and centrifuged again at 10,000× *g* for 10 min. Supernatants were stored until analysis. In brief, a working BCA reagent (4% copper sulfate solution diluted 1:50 in BCA reagent (both provided by Sigma–Aldrich)) was added to the samples. After incubating for 30 min at 37 °C in the dark, absorbance was measured at 550 nm using a microplate reader. Protein concentration was determined using a standard curve generated with 2 mg/mL BSA (Sigma–Aldrich).

### 2.4. Western Blot Analysis

Western blotting was employed to qualitatively analyze protein expression. Unless otherwise stated, all reagents and equipment were provided by Bio-Rad Laboratories, Inc. (Hercules, CA, USA). Following cell cultivation, cells were lysed with RIPA buffer and quantified as previously described. Each sample was mixed with 3.75 µL of 4x Laemmli buffer containing 10% 50 mM 2-mercaptoethanol (Thermo Fisher Scientific), heated at 95 °C for 5 min, and stored at −80 °C. Proteins (10–30 µg) were separated by SDS-PAGE on 8–15% polyacrylamide gels. A molecular weight marker (PageRuler™ Prestained Protein Ladder, Thermo Fisher Scientific) was included. Electrophoresis was performed at 80 V for 30 min and 120 V for 1.5 h. Proteins were transferred to a nitrocellulose membrane (Odyssey^®^, Li-Cor Biosciences, Lincoln, NB, USA) using BSN blotting buffer (48 mM Tris base and 39 mM glycine, 20% methanol (Th. Geyer GmbH & Co. KG)) for 17 h at 35 V and 4 °C. Membranes were stored in TBST buffer (10% 10× TBS at pH 7.6 (200 mM TRIS, 1500 mM NaCl in ddH_2_O), 0.1% Tween 20 diluted in ddH_2_O (Sigma–Aldrich)). Blotting success was assessed via Coomassie staining solution (0.05%, Rotiphorese^®^ Blau R, Carl Roth) for 30 min and subsequent heating. Gels were washed overnight in ddH_2_O, and the transfer assessment occurred the following day. For total protein normalization, the membranes were washed in ddH_2_O and stained with the Revert™ 700 Total Protein Stain Kit (Li-Cor Biosciences, Lincoln, NB, USA) according to the manufacturer’s protocol, with detection at 700 nm using the Odyssey Imager (with Image Studio™ Software 5.2 (LI-COR Biosciences)). Membranes were then destained with a Revert destaining solution (containing 30% methanol and 0.1 M NaOH 4N diluted in ddH_2_O) and blocked for 1 h at RT with Intercept^®^ (TBS) Blocking Buffer (LI-COR Biosciences). Primary antibodies (Table S2) were incubated overnight at 4 °C, followed by secondary antibodies (Table S3) for 1 h at RT in the dark. Detection was performed at 800 nm using the Odyssey Imager (Figure S1). For additional staining, membranes were stripped using NewBlot IR stripping buffer 5× (LI-COR Biosciences) according to the manufacturer’s instructions. Signal intensity was normalized to the total protein and the positive control.

### 2.5. Statistical Analysis

Experiments were conducted using varying numbers of biological replicates. For cell lines, different passages were used; for primary cells, different donors were employed, as indicated in the figure legends. GraphPad Prism 7 (GraphPad Software, San Diego, CA, USA) was used for all data visualization and statistical analyses. Data are expressed as means + standard deviations (SDs) of biological replicates (indicated as N in figure captions). One-way or two-way ANOVA was used for group comparisons, followed by appropriate post hoc tests as indicated. Statistical significance was defined as follows: * *p* < 0.05, ** *p* < 0.01, *** *p* < 0.001.

### 2.6. Usage of Generative AI and AI-Assisted Technologies

During the preparation of this work, the authors used ChatGPT 4.0 in order to improve readability. After using this tool or service, the authors reviewed and edited the content as needed and take full responsibility for the content of the publication.

## 3. Results

### 3.1. Proliferating Cell Models Express Minor Hepatic Differentiation Markers but Increased Tumor Markers

**Figure 1:**
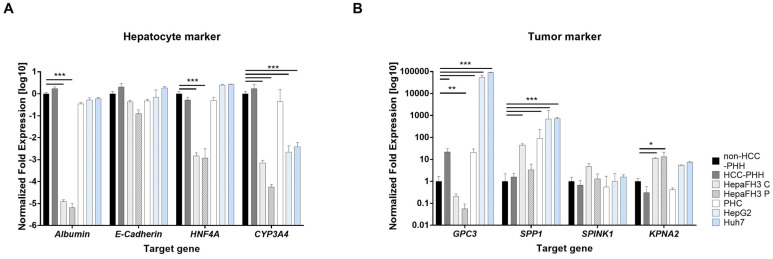
Transcript-level characterization of hepatic and tumor markers in various liver-derived cells. (**A**) Expression of hepatocyte differentiation markers—albumin, E-cadherin, hepatocyte nuclear factor 4 alpha (*HNF4A*), and cytochrome P450 3A4 (*CYP3A4*)—and (**B**) tumor-associated markers glypican-3 (*GPC3)*, secreted phosphoprotein 1 *(SPP1)*, serine peptidase inhibitor Kazal type 1 (*SPINK1)*, karyopherin subunit alpha 2 (*KPNA2)* were assessed by RT-qPCR in seven cell types: non-hepatocellular carcinoma primary human hepatocytes (non-HCC-PHHs, N = 5), hepatocellular carcinoma primary human hepatocytes (HCC-PHHs, N = 5), confluent HepaFH3 cells (HepaFH3 C, N = 3), proliferating HepaFH3 cells (HepaFH3 P, N = 3), primary human hepatoma cells (PHC, N = 5), HepG2 (N = 3), and Huh7 cells (N = 3). mRNA expression levels are presented as log10-transformed, normalized fold changes (means + standard deviation). Statistical analyses were performed on ΔCT values using two-way ANOVA followed by Tukey’s post hoc test. Significance thresholds were set at * *p* ≤ 0.05, ** *p* ≤ 0.01, *** *p* ≤ 0.001.

To distinguish metabolic features of liver regeneration from those of malignant transformation, we first examined marker profiles associated with hepatic differentiation and tumorigenesis. For this purpose, we compared proliferating hepatocytes (HepaFH3 cells) to non-proliferating PHHs, which serve as reference for healthy mature human liver cells. HCC was modeled using established HCLs (HepG2 and Huh7) as well as PHCs isolated from patient tissue. Transcript levels of key hepatic and tumor-associated markers were assessed using RT-qPCR (Figure 1A) to determine how closely the seven cell models reflected either regenerative or malignant states.

Both confluent and proliferating HepaFH3 cells (HepaFH3 C and HepaFH3 P) showed a statistically significant reduction in albumin, hepatocyte nuclear factor 4 alpha (*HNF4A*), and cytochrome P450 3A4 (*CYP3A4*) expression compared to non-HCC-PHHs isolated from tumor-free liver tissue. While the expression of albumin and *HNF4A* remained largely unchanged in HCC-PHHs, PHCs, and HCLs (HepG2 and Huh7), *CYP3A4* expression was significantly downregulated in the hepatoma cell lines. This decrease in albumin and *HNF4A* suggests that the HepaFH3 cells, regardless of their proliferative state, have reduced expression of key differentiation markers that are typically abundant in fully mature hepatocytes. Such a profile is often indicative of a dedifferentiated or more regenerative phenotype. Interestingly, E-cadherin expression remained consistent across all cell types, suggesting that epithelial characteristics were preserved even in metabolically or proliferatively altered cells.

In addition to hepatic differentiation markers, the expression of tumor-associated markers (*GPC3*, *SPP1*, *SPINK1*, and *KPNA2*) was examined across all cell models (Figure 1B). Glypican-3 (*GPC3*) and secreted phosphoprotein-1 (*SPP1*) were significantly upregulated in PHCs and HCLs (HepG2 and Huh7) compared to non-HCC-PHHs. In addition, *GPC3* expression was significantly increased in HCC-PHHs and *SPP1* expression was significantly increased in confluent HepaFH3 cells. Interestingly, *GPC3* was significantly downregulated in proliferating HepaFH3 cells, with a similar trend observed in the confluent state. These patterns suggest that while *GPC3* and *SPP1* are elevated in HCC-associated cells, *GPC3* expression is not driven by proliferation but rather reflects a diseased or pre-malignant state, particularly within the HCC context. Karyopherin subunit alpha 2 (*KPNA2*), however, was significantly elevated in both HepaFH3 cultivation states and showed a trend toward increased expression in HCLs, though this was not statistically significant. This pattern suggests that *KPNA2* may be more reflective of cellular proliferation than of malignant transformation.

Taken together, these results imply that several classic tumor markers are also detectable in non-malignant proliferating cells, indicating their potential role as proliferation markers rather than exclusive indicators of cancer.

### 3.2. Upcyte^®^ Hepatocytes Display a Similar Expression of Metabolic Genes to Hepatoma Cell Lines

**Figure 2:**
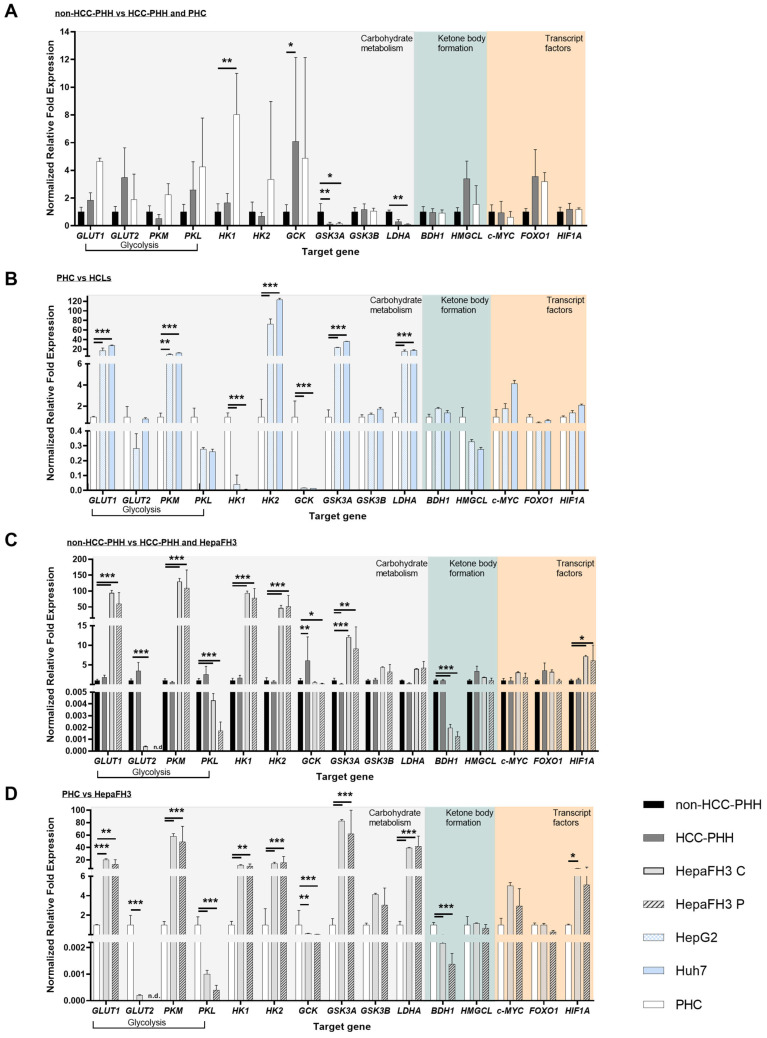
Transcript-level analyses of energy metabolism-related genes in liver-derived cell types. RT-qPCR was used to quantify expression of genes involved in glycolysis, carbohydrate metabolism, ketone body formation, and transcriptional regulation. Cells were either cultured or snap-frozen (primary human hepatoma cells (PHCs)) following isolation. (**A**) Comparison of non-hepatocellular carcinoma primary human hepatocytes (HCC-PHHs, N = 5), HCC-derived primary human hepatocytes PHHs (HCC-PHHs, N = 5), and PHCs (N = 5). (**B**) Comparison of PHCs, (N = 5) with hepatoma cell lines (HCLs): HepG2 (N = 3) and Huh7 (N = 3). (**C**) Hepatocytes (non-HCC-PHHs, N = 5) were used as a basis to determine the differential gene expression of HCC-PHHs (N = 5) and Upcyte^®^ hepatocytes in confluent (HepaFH3 C, N = 3) and proliferating (HepaFH3 P, N = 3) conditions. (**D**) PHCs (N = 3) were compared to HepaFH3 C (N = 3), as well as HepaFH3 P (N = 3). Gene expression values (n = 3) are presented as mean + standard deviation (SD), shown as normalized relative fold expression, n.d. meaning not detected. Statistical analyses were performed on ΔCT values using two-way ANOVA followed by Dunnett’s post hoc test. The significance levels were set at * *p* ≤ 0.05, ** *p* ≤ 0.01, and *** *p* ≤ 0.001. Genes analyzed include *BDH1*, 3-hydroxybutyrate dehydrogenase 1; *c-MYC*, MYC proto-oncogene; *FOXO1*, forkhead box O1; *GCK*, glucokinase; *GLUT1*, glucose transporter type 1; *GLUT2*, glucose transporter type 2; *GSK3A*, glycogen synthase kinase 3 alpha; *GSK3B*, glycogen synthase kinase 3 beta; *HIF1A*, hypoxia-inducible factor 1 alpha; *HK1*, hexokinase 1; *HK2*, hexokinase 2; *HMGCL*, 3-hydroxymethyl-3-methylglutaryl-CoA lyase; *LDHA*, lactate dehydrogenase A; *PKL*, pyruvate kinase L; *PKM*, pyruvate kinase M1/2.

To better understand the metabolic characteristics of the different hepatic cell models, a detailed transcript-level analyses of genes involved in glucose metabolism, ketone body utilization, and associated regulatory transcription factors were performed. Transcript levels of enzymes and regulators were assessed across all cell models and grouped according to their functional role in energy-related pathways (Figure 2). Four pairwise comparisons were carried out: (Figure 2A) primary hepatic cells (non-HCC-PHHs, HCC-PHHs, and PHCs), (Figure 2B) hepatoma cell lines (HepG2 and Huh7) compared to PHCs, (Figure 2C) HepaFH3 cells (confluent and proliferating) compared to non-HCC-PHHs and HCC-PHHs, and (Figure 2D) HepaFH3 cells compared to PHCs. This approach allowed us to distinguish proliferation- and cancer-associated metabolic profiles from normal hepatic physiology.

Compared to non-HCC-PHHs, both HCC-PHHs and PHCs exhibited selected alterations in the expression of genes involved in energy metabolism (Figure 2A). PHCs showed a significant upregulation of *HK1* and significant downregulation of *LDHA*, while *GSK3A* was significantly downregulated in both PHCs and HCC-PHHs. Additionally, *GCK* was slightly but significantly upregulated in HCC-PHHs. These findings point to subtle but distinct transcriptional changes in key metabolic enzymes. Although other genes related to glycolysis, such as *GLUT1* and *PKM*, and transcriptional regulators like *FOXO1* showed observable trends in PHCs, these differences did not reach statistical significance.

Compared to PHCs, the hepatoma cell lines HepG2 and Huh7 exhibited a significant upregulation of glycolysis-related genes, including *GLUT1* and *PKM* (Figure 2B). In addition, the expression of *HK2*, *GSK3A*, and *LDHA* were significantly elevated in HCLs, indicating enhanced glycolytic activity and altered carbohydrate metabolism. In contrast, *HK1* and *GCK*—both associated with hepatocyte-specific glucose processing—were significantly downregulated in both cell lines.

To assess the metabolic characteristics of non-malignant proliferating hepatocytes, we compared the transcript profiles of HepaFH3 cells and PHHs, with statistical significance calculated relative to non-HCC-PHHs (Figure 2C). Both confluent and proliferating HepaFH3 cells showed a distinct shift toward a glycolytic phenotype, with significantly increased expression of *GLUT1*, *PKM*, *HK1*, *HK2*, and *GSK3A*. Concurrently, genes associated with hepatocyte-specific and oxidative metabolism, including *PKL* and 3-hydroxybutyrate dehydrogenase 1 (*BDH1*), were significantly downregulated in both HepaFH3 states. Additionally, the transcription factor *HIF1A* was mildly but significantly upregulated. In addition, *GLUT2* was significantly downregulated in confluent HepaFH3 cells, whereas *GCK* was observed to be significantly downregulated in the proliferating cells. This metabolic signature was more pronounced in proliferating cells, reflecting increased energy demands during cell cycle activity.

A comparison of PHCs with HepaFH3 cells revealed differences in the expression of genes related to glycolysis, carbohydrate metabolism, ketone body utilization, and transcriptional regulation (Figure 2D). Both confluent and proliferating HepaFH3 cells showed significantly elevated expression of *GLUT1*, *PKM*, *HK1*, *HK2*, *GSK3A*, and *LDHA* compared to PHCs. *HIF1A* expression was significantly increased in the confluent state and showed a trend toward upregulation in proliferating cells. In contrast, the expression of *GLUT2*, *PKL*, *GCK*, and *BDH1* was significantly reduced in both HepaFH3 cultivation states. These consistent differences suggest that the metabolic profile of HepaFH3 cells reflects proliferation-associated reprogramming distinct from the metabolic phenotype of malignant hepatocytes.

Taken together, the transcript data reveal that metabolic reprogramming occurs across the spectrum of hepatic cell models but follows distinct patterns depending on the biological context. While PHCs exhibit a partial metabolic shift, HCLs show a more pronounced Warburg-like phenotype characterized by enhanced glycolysis, lactate secretion, mitochondrial activity, and loss of hepatic identity. In contrast, HepaFH3 cells—despite being non-malignant—display a robust proliferation-associated metabolic signature, including upregulation of glycolytic enzymes and suppression of hepatocyte-specific metabolic genes.

### 3.3. Tumor Metabolism Correlates with Proliferation

**Figure 3:**
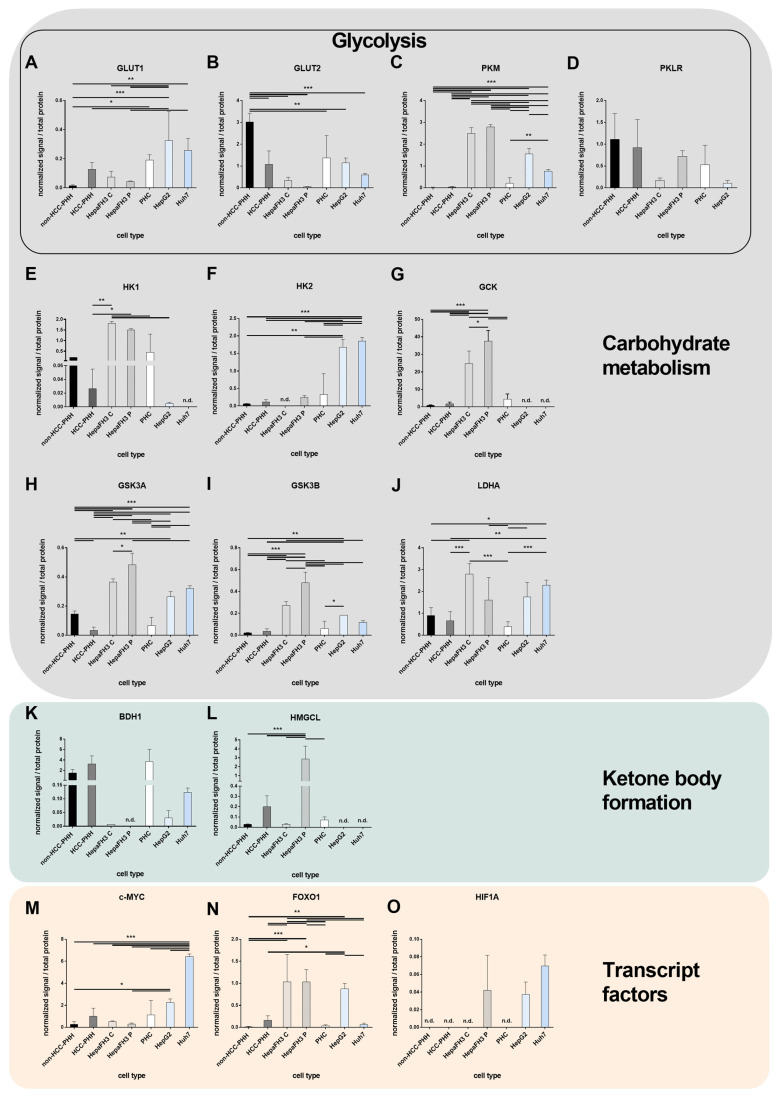
Protein-level analyses of energy metabolism-related pathways in liver-derived cell types. Cells were either cultured or snap frozen (primary human hepatoma cells, PHCs) immediately following isolation, and Western blot analyses were performed to quantify protein expression (Figure S1). Expression of relevant proteins involved in (**A**–**J**) glycolytic pathways, (**K**,**L**) ketone body formation, and (**M**–**O**) transcript factor regulation is shown. Samples included non-HCC primary human hepatocytes (non-HCC-PHHs, N = 5), HCC-derived PHHs (HCC-PHHs, N = 5), PHCs (N = 5), Upcyte^®^ hepatocytes HepaFH3 cells in confluent (HepaFH3 C, N = 3) and proliferating (HepaFH3 P, N = 3) states, and hepatoma cell lines (HCLs: HepG2 (N = 3) and Huh7 (N = 3)). Proteins analyzed include 3-hydroxybutyrate dehydrogenase 1 (BDH1), MYC proto-oncogene (c-MYC), forkhead box O1 (FOXO1), glucokinase (GCK), glucose transporter type 1 and 2 (GLUT1, GLUT2), glycogen synthase kinase 3 alpha and beta (GSK3A, GSK3B), hypoxia-inducible factor 1 alpha (HIF1A), hexokinases (HK1, HK2), 3-hydroxymethyl-3-methylglutaryl-CoA lyase (HMGCL), lactate dehydrogenase A (LDHA), and pyruvate kinases (PKL, PKM). Some proteins were not detected (n.d.). Data are presented as mean + standard deviation (SD). Statistical analyses were performed using one-way ANOVA followed by Tukey’s post hoc test. Significance levels were set at * *p* ≤ 0.05, ** *p* ≤ 0.01, *** *p* ≤ 0.001.

To validate transcript-level findings, selected targets of energy metabolism were also examined at the protein level, including markers of glycolysis, ketone metabolism, I appreciate your attention to this detail. Nonetheless, the single and double lines require no further explanation in the figure captions. They refer to the statistical differences between two groups studied.and metabolic transcript factors (Figure 3).

The glucose transport protein GLUT1 (Figure 3A) showed a low expression in non-malignant cells (PHHs and HepaFH3) but was markedly upregulated in HCLs and moderately elevated in PHCs. GLUT2 (Figure 3B) was progressively downregulated with increasing proliferative capacity, particularly in HCLs, PHCs, and HepaFH3 cells. Pyruvate kinase (PKM, Figure 3C) showed low expression in primary cells but was significantly upregulated in HepaFH3, HepG2, and Huh7 cells. The liver-specific isoform PKLR (Figure 3D) exhibited relatively uniform expression across all cell types. HK1 (Figure 3E) levels were low in HCLs, whereas HK2 (Figure 3F), often associated with tumor metabolism, was significantly upregulated in HCLs compared to all primary cells but remained low in HepaFH3. GCK (Figure 3G) was notably elevated in HepaFH3 cells, with significantly higher expression in proliferating versus confluent cultures.

The protein profiles of GSK3 isoforms (Figure 3H,I) revealed strong induction in HepaFH3 cells, particularly under proliferating conditions. Both GSK3A and GSK3B were significantly upregulated in HepaFH3 P cells compared to their confluent counterparts and to all primary cell types, including PHCs and PHHs. Elevated expression was also observed in HCLs (HepG2 and Huh7), although at lower levels than in HepaFH3 P cells. LDHA (Figure 3J), a key enzyme in anaerobic glycolysis and an important marker of the Warburg effect, was significantly elevated in confluent HepaFH3 cells and HCLs compared to all primary cell types. In proliferating HepaFH3 cells, LDHA was only modestly increased, with significant upregulation observed only in comparison to PHCs.

For ketone metabolism, BDH1 protein levels (Figure 3K) exhibited no significant differences across all cell types; however, a downward trend was observed in HepaFH3 cells. This phenomenon can also be observed at the transcript level. In contrast, 3-hydroxymethyl-3-methylglutaryl-CoA lyase (HMGCL) (Figure 3L) showed a proliferation-specific increase, with significantly elevated expression detected only in HepaFH3 cells under proliferating conditions.

The transcription factors c-MYC, FOXO1, and HIF1A displayed distinct expression patterns across the cell models (Figure 3M–O). C-MYC (Figure 3M) expression was significantly elevated in HCLs, HepG2 and Huh7, compared to primary liver cells and HepaFH3 cells. In contrast, PHCs exhibited a high donor variability with average expression levels comparable to those in HepG2 cells. FOXO1 (Figure 3N) was significantly upregulated in both confluent and proliferating HepaFH3 cells, as well as in HepG2 cells, suggesting a link to proliferation-associated signaling. HIF1A expression (Figure 3O) was detectable only in proliferating HepaFH3 cells and in both HCLs, but no statistically significant differences were observed among these groups, indicating that its protein levels may be post-transcriptionally regulated or conditionally expressed.

In summary, protein-level analyses confirmed key aspects of the transcript-level findings, particularly for glycolytic enzymes and regulators such as GLUT1, PKM, HK1, and GSK3A/B, which showed consistent upregulation in proliferating and malignant models. Notably, the protein expression patterns helped to differentiate non-malignant proliferative cells from tumor-derived models, most clearly through the selective upregulation of HMGCL in HepaFH3 cells and c-MYC in HCLs. While most trends were in agreement with transcript data, some discrepancies—such as those observed for BDH1, GCK, and FOXO1—suggest additional layers of regulation at the post-transcriptional or translational level.

### 3.4. Liver Cells Adjust Their Energy Metabolism in Response to Proliferation

**Figure 4:**
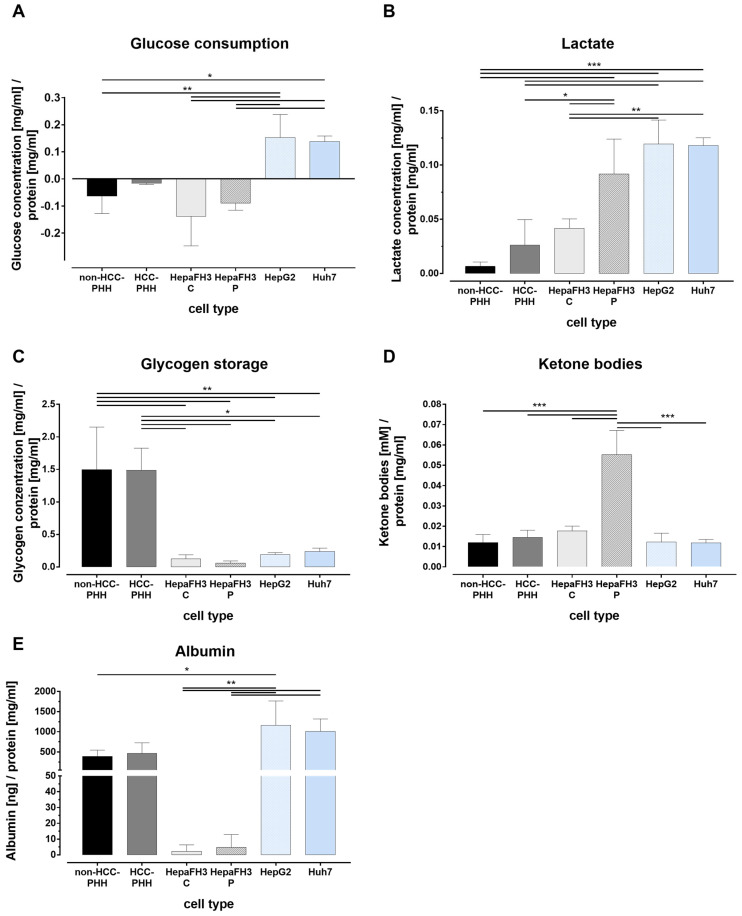
Functional metabolic profiling of proliferating and non-proliferating liver cell types. Primary human hepatocytes not derived from hepatocellular carcinoma (non-HCC-PHHs, N = 5), HCC-derived primary human hepatocytes (HCC-PHH, N = 2), Upcyte^®^ HepaFH3 hepatocytes in confluent (HepaFH 3 C, N = 3) and proliferating (HepaFH3 P, N = 3) states, and hepatoma cell lines (HCLs: HepG2 (N = 3) and Huh7 (N = 3)) were analyzed for key metabolic parameters: (**A**) glucose consumption, (**B**) lactate secretion, (**C**) glycogen storage, (**D**) ketone body production, and (**E**) albumin secretion. PHCs were excluded from functional assays due to technical limitations in culturing. All values were normalized to total protein content and are presented as mean + standard deviation (SD). Statistical significance was determined using one-way ANOVA followed by Tukey’s post hoc test. Significance thresholds were set at * *p* ≤ 0.05, ** *p* ≤ 0.01, and *** *p* ≤ 0.001 representing statistical significance.

To complement the analyses of transcript data and protein expression, we performed functional metabolic assays across six hepatic cell models (Figure 4). These included primary hepatocytes from non-HCC and HCC donors, confluent and proliferating Upcyte^®^ HepaFH3 cells, and the hepatoma cell lines HepG2 and Huh7. Glucose levels in the culture medium were measured to assess uptake or production (Figure 4A). Non-HCC-PHHs, HCC-PHHs, and HepaFH3 cells did not show net glucose uptake, in contrast to HCLs, with the strongest uptake observed in tumor cell lines. Lactate accumulation after a four-hour starvation period served as a readout for glycolytic activity (Figure 4B). Lactate levels were significantly elevated in both HepaFH3 cultivation states as well as in HepG2 and Huh7 cells, consistent with increased glycolytic flux and a shift toward anaerobic energy metabolism. Glycogen storage capacity differed markedly across models (Figure 4C). Non-HCC-PHHs and HCC-PHHs retained the highest glycogen levels, with some donor-dependent variability (Figure S2). In contrast, glycogen content was significantly reduced in HepaFH3 cells and HCLs, indicating a loss of liver-specific storage function in proliferative and malignant contexts. Ketogenesis was assessed by quantifying β-HB as a marker for ketone body production (Figure 4D). A significant increase was observed only in the proliferating HepaFH3 cells, while all other models, including primary hepatocytes and tumor cell lines, showed comparably low levels of ketone body synthesis. To confirm hepatic differentiation, we also measured albumin secretion (Figure 4E). While HCC-PHHs, HepG2 and Huh7 cells showed comparable albumin levels, HepaFH3 cells exhibited a significant reduced albumin secretion, regardless of cultivation mode, suggesting a partial dedifferentiation of this cell model.

Taken together, these results confirm that proliferating cells undergo metabolic reprogramming characterized by increased glycolysis, reduced glycogen storage, and altered ketone metabolism. These functional adaptations closely mirror the changes at the transcript and protein levels and support a strong link between metabolic remodeling and proliferative activity in hepatic cells. These functional results validate the transcript and protein findings, particularly for glycolytic activation, glycogen depletion, and reduced hepatocyte-specific functions in proliferative and malignant models. The increased ketone body production in HepaFH3 P further supports a unique metabolic profile associated with regenerative proliferation.

## 4. Discussion

The present study aimed to characterize the metabolic signatures of diverse hepatic cell models representing different stages of hepatocellular proliferation and malignant transformation. Hepatocellular carcinoma remains one of the most common and lethal cancers worldwide [6], and metabolic rewiring is increasingly recognized as a hallmark of both tumor progression and cell proliferation [11,51,52,53].

Our previous work showed that proliferating HepaFH3 cells share key metabolic traits with HCLs, including increased glucose uptake and lactate secretion, supporting their use as a surrogate model for regenerative or pre-malignant hepatocytes [19]. Building on our previously published work, which critically evaluated the usability of HCLs HepG2 and Huh7 as models for resectable HCC [13], we extended the scope by incorporating non-malignant proliferating hepatocytes (HepaFH3) paired with primary cells from HCC patients and functional metabolic readouts. Our central hypothesis, that metabolic reprogramming overlaps between regeneration and malignancy, was tested by integrating investigations at the mRNA, protein, and metabolite levels across seven hepatic models. The resulting data allowed us to delineate both shared and distinct features of regenerative and cancer-associated metabolic adaptations.

Assessment of hepatic identity markers revealed a consistent dedifferentiation pattern in both malignant and regenerating proliferative cells. Transcript levels of classic hepatocyte markers, albumin and *HNF4A*, were significantly reduced in HepaFH3 cells, and *CYP3A4* was expressed equally low in both HepaFH3 and HCLs, consistent with previously reported downregulation of hepatic differentiation markers in both HCC and proliferating in vitro models [50,54,55]. These findings are also in line with reports showing reduced hepatic function in Upcyte^®^ hepatocytes under proliferative conditions [56,57]. Notably, albumin protein levels and functionally measured glycogen storage capacity were also decreased in HepaFH3 cells, which is consistent with a loss of hepatic-specific metabolic function and reduced maturity [19]. These results highlight that dedifferentiation is not exclusive to cancer cells but also occurs during regenerative proliferation, making it an unreliable standalone indicator of malignancy.

Tumor-associated marker profiles showed both overlapping and distinct expression patterns across models. *GPC3* and *SPP1*, well-established markers of HCC [58,59], were significantly upregulated in both PHCs and HCLs but also detected at moderate levels in HCC-PHHs and HepaFH3 cells. Similarly, serine peptidase inhibitor Kazal type 1 (*SPINK1*)—often associated with hepatic proliferation and cancer [60,61]—was elevated across several models, indicating limited specificity for malignancy. In contrast, *KPNA2* was predominantly increased in proliferative models, including HepaFH3, supporting its association with cell cycle regulation rather than malignant transformation per se [62,63].

Interestingly, subgroup analyses of paired PHCs and HCC-PHHs, which were obtained from resected HCC patient samples with associated clinical metadata, revealed a moderate inverse correlation between *GPC3* expression and patient α-fetoprotein (AFP) serum levels (Table 1) in some cases, aligning with reports that *GPC3* expression may be elevated in AFP-negative HCC patients [64] (Figure S3). These patterns suggest that many so-called tumor markers also reflect cellular proliferation and metabolic activation, underscoring the need for multi-parameter approaches when differentiating between regenerative and malignant states. While our study included primary human HCC and adjacent non-tumorous liver tissue, clinical metadata such as AFP levels, tumor stage, and histological grading were available only for a subset of patients and not integrated into the molecular analyses. The absence of blood samples further limited metabolic correlation. Given the small cohort size and heterogeneity, robust clinical associations were not feasible. However, preliminary trends (e.g., inverse relationship between *GPC3* and AFP; Figure S3) suggest potential clinical relevance. Future studies with larger, well-annotated cohorts and matched plasma metabolomics will be required to validate candidate metabolic markers in a translational setting. Transcript-level analyses revealed a consistent upregulation of key glycolytic enzymes—including *GLUT1*, *PKM*, *HK1*, *HK2*, and *GSK3A*—in both proliferating HepaFH3 cells and HCLs (HepG2, Huh7), compared to non-HCC-PHHs. These changes reflect a transcriptional reprogramming toward glycolytic metabolism, consistent with the Warburg effect, and were functionally validated by elevated glucose consumption and lactate secretion [12,19]. In contrast, genes linked to hepatocyte-specific glucose metabolism, including *GLUT2, GCK*, and *PKL*, were significantly downregulated in HepaFH3 cells, suggesting metabolic dedifferentiation. The canonical switch from *GCK* to *HK2*, a known hallmark of HCC progression [21,22,24], was only observed in HCLs. In contrast, HepaFH3 cells showed high transcriptional *HK2* expression, but maintained *GCK* expression in the confluent state, with *GCK* being downregulated in the proliferating state. The metabolic profile of HepaFH3 cells, characterized by *HK2* expression and proliferation-dependent modulation of *GCK*, indicates a non-malignant proliferative phenotype. Nonetheless, comparing the transcript data with the protein data revealed notable discrepancies for key metabolic enzymes such as HK2, GCK, and BDH1. mRNA and protein levels often correlate only modestly, probably due to post-transcriptional regulation, including translation control and protein turnover [65]. Interestingly, BDH1 protein levels remained low despite detectable transcript expression in HepaFH3 cells, suggesting possible post-translational degradation. While BDH1 is known to be downregulated in HCC at the mRNA and protein levels [66,67], to our knowledge, no studies to date have identified specific molecular mechanisms—such as ubiquitination, phosphorylation, or regulated proteasomal degradation—that selectively govern BDH1 protein stability. Additionally, the discordance between HK2 and GCK transcript vs. protein may be mediated by region- or context-specific miRNAs, such as liver-enriched miR-122 or miR-155, which are known to modulate glycolytic enzyme expression [68,69]. Such regulatory mechanisms are especially important in proliferating or stressed cells, where metabolic enzyme abundance must be rapidly adjusted to meet changing demands. Our findings underscore the necessity of integrating functional analyses with transcript- and protein-level data expression to accurately interpret metabolic regulation in liver cell models.

Interestingly, *HIF1A*, a central transcription regulator of hypoxia-induced glycolysis, was significantly upregulated only in proliferating HepaFH3 cells but not in PHCs or HCLs at the transcript level, suggesting differential regulation of anaerobic glycolysis and a metabolic adaptation for higher proliferation. Protein-level analyses of transcription factors supported this distinction: c-MYC, a major oncogene and driver of hepatocarcinogenesis [70,71,72], was exclusively elevated in HCLs, whereas proliferating HepaFH3 cells showed high HIF1A but minimal c-MYC expression. HIF1A is known to promote the expression of glycolytic enzymes such as LDHA, HK2, and GLUT1 [34,35], while c-MYC not only enhances glycolysis but also regulates cell fate, apoptosis, and differentiation [73,74]. These findings suggest that proliferative metabolic remodeling in HepaFH3 is primarily HIF1A-driven, whereas malignant reprogramming in HCLs involves both HIF1A and c-MYC. Supporting this interpretation, *GPC3* and c-MYC showed parallel upregulation in HCLs but not in HepaFH3 or PHHs, consistent with reports that c-MYC can transcriptionally upregulate *GPC3* expression, reinforcing their potential combined value as tumor biomarkers [75].

Beyond glycolytic remodeling, we examined ketone body metabolism as a complementary energy pathway with potential diagnostic value. In the liver, ketone bodies such as β-HB and acetoacetate are synthesized from fatty acids during nutrient deprivation or fasting states. These are then exported to peripheral tissues for use as alternative energy substrates [76]. Hepatocytes typically lack the capacity to oxidize ketone bodies themselves, due to the absence of key enzymes required for ketolysis, including OXCT1 [77]. However, in HCC, reactivation of ketone body metabolism has been reported, with increased expression of ketolytic enzymes enabling tumor cells to scavenge alternative energy sources under metabolic stress [36,78]. For instance, Huang et al. demonstrated that BDH1, OXCT1 activity, and ketone utilization in HCC cells is induced by mTORC2-AKT-SP1 signaling under starvation conditions [36].

In our study, we observed a marked increase in total ketone body levels, particularly β-HB in proliferating HepaFH3 cells, accompanied by elevated expression of HMGCL. This suggests enhanced ketogenesis under non-malignant proliferative conditions.

In contrast, BDH1 expression remained low at both transcript and protein levels. The absence of ketone body utilization may reflect a lack of key signaling pathways, such as the mTORC2–AKT–SP1 axis [36], which is known to promote BDH1 expression and ketone metabolism in malignant cells.

Beyond this, it is also plausible that β-HB accumulation in HepaFH3 cells reflects a redox-regulatory overflow mechanism, analogous to lactate formation during aerobic glycolysis. One hypothesis emerging from our data is that the conversion of acetoacetate to β-HB, even at low BDH1 activity, contributes to NAD^+^ regeneration under high glycolytic or β-oxidative activity. This would help maintain redox homeostasis and support biosynthetic flux during proliferation. This concept is consistent with studies showing that the β-HB/acetoacetate ratio [79] reflects mitochondrial NAD^+^/NADH status and that ketone metabolism is linked to redox-sensitive regulatory networks [80].

A second hypothesis is that ketogenesis may also serve as a metabolic rescue mechanism to prevent the accumulation of excess mitochondrial acetyl-CoA. Under conditions of high β-oxidation or limited TCA cycle capacity, ketone body formation allows the regeneration of free CoA from acetyl-CoA, thereby sustaining mitochondrial flux. This concept of “Coenzyme A economy” has been described as an essential mechanism for maintaining hepatic energy homeostasis under catabolic conditions [81,82,83] and may be particularly relevant in proliferating HepaFH3 cells, where metabolic demand is elevated but full oxidative capacity may be constrained. The accumulation of β-HB observed in our study could thus reflect not only impaired ketolysis but also the use of ketogenesis as a CoA-sparing pathway.

Taken together, the observed ketone metabolism profile in HepaFH3 cells likely reflects both the absence of malignant ketolytic signaling and a redox-driven compensatory mechanism. In contrast, malignant models such as PHCs and HCLs exhibited higher BDH1 expression and may possess the metabolic flexibility to utilize ketone bodies. While these findings suggest distinct metabolic routing of ketone bodies in regenerative versus malignant contexts, functional assays (e.g., isotope tracing) will be required to confirm this working model.

The integration of transcript, protein, and metabolite levels underscores the complex and layered regulation of metabolism in hepatic cell models. While metabolic remodeling was a consistent feature of proliferative states, it was not specific to malignant transformation. The HepaFH3 model recapitulated many features of tumor metabolism, including glycolytic activation and dedifferentiation, yet lacked key transformation markers such as c-MYC- and BDH1-driven ketone utilization. HepaFH3 cells were generated from PHHs via lentiviral transduction to extend proliferation capacity. These cells retain hepatic functions, display no chromosomal abnormalities, and lack tumorigenic features, supporting their classification as non-malignant and reliable for metabolic studies [84]. Moreover, the selective upregulation of HIF1A in proliferative cell models and the distinct induction of ketogenesis via HMGCL highlight metabolic adaptations specific to regenerative proliferation. These findings illustrate the limitations of interpreting metabolic traits in isolation and emphasize the need for context-aware, multi-marker assessments to distinguish regenerative from malignant reprogramming.

Taken together, our findings underscore the importance of metabolic profiling in hepatic research and demonstrate that proliferation-associated metabolic traits can mimic malignancy without indicating transformation. The HepaFH3 model, particularly in its proliferative state, emerges as a robust surrogate for studying regenerative metabolism, while highlighting the need for multi-parametric approaches to distinguish regeneration from cancer. These insights lay the groundwork for improved model selection and may contribute to more accurate diagnostic and therapeutic strategies in liver disease and HCC.

This study has several limitations that should be acknowledged. First, while HepaFH3 cells represent a proliferative, non-malignant hepatocyte model, they are derived from a single donor and genetically modified via lentiviral transduction. This may limit the generalizability of findings and introduce alterations not reflective of native regeneration. Second, PHHs and PHCs were isolated from a limited number of donors, and inter-donor variability could have influenced transcript expression levels and functional outcomes. Additionally, the low availability and phenotypic heterogeneity of PHC donors may limit the generalizability of comparisons with malignant hepatocytes. Third, PHCs were excluded from functional assays due to low plating efficiency, restricting direct functional comparisons between malignant and non-malignant primary models. Fourth, we observed some discrepancies, between mRNA and protein expression (e.g., *FOXO1*, *BDH1*, *HK2*, *GCK*), likely due to post-transcriptional regulation or protein turnover. Fifth, functional assays were conducted as endpoint measurements with small sample sizes, which limits kinetic insight and reduces statistical power, particularly for donor-derived PHHs and PHCs. This reflects the broader challenge of obtaining sufficient matched primary hepatocytes and tumor cells from the same HCC patient, a bottleneck that significantly limits sample availability for translational studies. Finally, although we provide detailed metabolic characterization, we did not directly evaluate how these metabolic shifts affect proliferation or viability under metabolic stress. Additional functional studies under stress conditions are needed to establish causal relationships. However, many of our findings were consistent with previously published work. Our observations of glucose uptake, lactate accumulation, dedifferentiation markers, and glycolytic enzyme regulation in HepaFH3 and HCLs are in agreement with results from Scheffschick et al. and Schicht et al., supporting the reproducibility of key metabolic trends across different studies and experimental setups [13,19]. Importantly, this consistency across independent datasets adds confidence to our conclusions, even in the face of donor variation and technical limitations.

## 5. Conclusions

This study highlights significant metabolic alterations across various in vitro liver cell models. Proliferating HepaFH3 cells exhibited low expression of hepatic differentiation markers, increased glycolytic activity, and ketone body accumulation, consistent with a metabolic shift toward proliferation rather than differentiation, without evidence of malignant transformation. Key markers such as *GPC3* and c-MYC were validated as indicators of tumorigenesis, with *GPC3* showing relevance in AFP-negative HCC. The metabolic profile of HepaFH3 cells diverges from both non-HCC-PHHs and malignant cells (PHCs and HCLs), underscoring that metabolic reprogramming is not exclusive to malignancy but also occurs in regenerative contexts. While HepaFH3 cells do not fully recapitulate either cancerous or fully differentiated non-cancerous hepatic states, they represent a distinct and valuable in vitro model for studying regeneration-associated metabolic changes.

Looking ahead, future studies should incorporate comprehensive omics profiling to validate our findings on a broader scale, along with extended culture durations and the modeling of stress conditions (e.g., hypoxia or nutrient deprivation) to better replicate in vivo liver physiology. Furthermore, expanding the PHC cohort to include donors with a broader range of HCC stages and histological grades would strengthen the clinical relevance of model comparisons. Such extensions will be essential for refining our understanding of the metabolic overlap and distinctions between regeneration and malignancy, and for enhancing the translational potential of in vitro liver models.

## Figures and Tables

**Table 1:** Clinical and pathological parameters of patients used for PHH and PHC isolation.

Donor	Age	Sex	Diagnosis	BMI	Steatosis [%]	ASH	NASH	Fibrosis	Cirrhosis	AFP [ng/mL]
D1	72	F	iCCA	29	5	no	no	minor portal	none	-
D2	49	M	Caroli disease	29	30	no	yes	minor portal	none	-
D3	38	M	NET	28	<1	no	no	minor portal	none	-
D4	45	F	FNH	19	none	no	no	none	none	-
D5	59	F	PLD	24	N/A	N/A	N/A	minor–moderate	N/A	-
DH1/DC1	45	F	HCC rez. G2	18	2	no	no	minor	none	149
DH2/DC2	63	M	HCC G1	34	5	no	no	minor–moderate	none	2.57
DH3/DC3	66	M	HCC G1	28	5	no	no	minor	none	263
DH4/DC4	79	F	HCC G1	28	60	no	yes	moderate–high	minor–active	0
DH5/DC5	74	M	HCC G2	26	50	no	no	minor–moderate	none	1657

Non-HCC-PHHs (D1–D5); HCC-PHHs (DH1–DH5); PHCs (DC1–DC5); G1, G2 grading of HCC according to the WHO [38]. Abbreviations: ASH, alcoholic steatohepatitis; BMI, body mass index; F, female; FNH, focal nodular hyperplasia; HCC, hepatocellular carcinoma; iCCA, intrahepatic cholangiocarcinoma; M, male; NASH, non-alcoholic steatohepatitis; N/A, not available; NET, neuroendocrine tumor; PLD, polycystic liver disease.

**Table 2:** RT-qPCR primer pairs.

Gene Name	Forward	Reverse
albumin	TTGATTGCCTTTGCTCAGTA	GCCATTTCACCATAGGTTTC
*GPC3*	TGTGCCCATTCTCAACAACG	AGCAAAGGGTGTCGTTTTCC
*KPNA2*	AGGAAAACCGCAACAACCAG	TTTCGGAATCAAACCAGCCC
*SPINK1*	AGAGACGTGGTAAGTGCGG	ATTTGGCCTCTCTTCCCAGG
*SPP1*	CACACATGGAAAGCGAGGAG	TGGAATTCACGGCTGACTTTG

The primers were designed in-house and purchased from biomers.net GmbH. They were dissolved in nuclease-free water at a 1:10 ratio. Abbreviations: *GPC3*, glypican-3; *KPNA2*, karyopherin subunit alpha 2; *SPINK1*, serine peptidase inhibitor Kazal type 1; *SPP1*, secreted phosphoprotein 1.

## Data Availability

The data presented in this study are available on request from the corresponding author.

## Supplementary Materials

The following supporting information can be downloaded at: https://www.mdpi.com/article/10.3390/cells14161254/s1, Table S1: Gene-specific primers purchased from Qiagen (Hilden, Germany) for RT-qPCR analyses.; Table S2: Primary antibodies used for Western blot analyses.; Table S3: Secondary antibodies used for Western blot analyses.; Figure S1: Expression of various proteins in primary hepatic cells and hepatic cell lines.; Figure S2: Glycogen levels in PHHs from non-HCC and HCC donors.; Figure S3: *GPC3* gene expression and AFP serum levels in HCC-PHHs and corresponding PHCs.

## Abbreviations

The following abbreviations are used in this manuscript:

AFP	α-fetoprotein
ASH	alcoholic steatohepatitis
BDH1	3-hydroxybutyrate dehydrogenase
β-HB	β-hydroxybutyrate
BMI	body mass index
BSA	bovine serum albumin
CUP	cancer of unknown primary
CYP	cytochrome P450
c-MYC	MYC proto-oncogene
EGTA	ethylene glycol tetraacetic acid
ELISA	enzyme-linked immunosorbent assay
F	female
FNH	focal nodular hyperplasia
FOXO1	forkhead box O1
GCK	glucokinase
GLUT	glucose transporter
*GPC3*	glypican-3
GSK3	glycogen synthase kinase 3
HCC	hepatocellular carcinoma
HCL	hepatoma cell line
HepaFH3 C	HepaFH3 cells confluent
HepaFH3 P	HepaFH3 cells proliferating
HIF1A	hypoxia-inducible factor 1 subunit alpha
HK	hexokinase
HMGCL	3-hydroxymethyl-3-methylglutaryl-CoA lyase
*HNF4A*	hepatocyte nuclear factor 4 alpha
iCCA	intrahepatic cholangiocarcinoma
*KPNA2*	karyopherin subunit alpha 2
LDHA	lactate dehydrogenase A
M	male
MASLD	metabolic dysfunction-associated fatty liver disease
N/A	not available
NASH	non-alcoholic steatohepatitis
NET	neuroendocrine tumor
PHC	primary human hepatoma cell
PHH	primary human hepatocytes
PKL	pyruvate kinase liver
PKM	pyruvate kinase M1/2
PLD	polycystic liver disease
*SPINK1*	serine protease inhibitor Kazal type 1
*SPP1*	secreted phosphoprotein-1

## References

[1] Michalopoulos G.K. (2007). Liver regeneration. J. Cell. Physiol..

[2] Fausto N., Campbell J.S., Riehle K.J. (2006). Liver regeneration. Hepatology.

[3] Solhi R., Lotfinia M., Gramignoli R., Najimi M., Vosough M. (2021). Metabolic hallmarks of liver regeneration. Trends Endocrinol. Metab. TEM.

[4] Vander Heiden M.G., Cantley L.C., Thompson C.B. (2009). Understanding the Warburg effect: The metabolic requirements of cell proliferation. Science.

[5] DeBerardinis R.J., Lum J.J., Hatzivassiliou G., Thompson C.B. (2008). The biology of cancer: Metabolic reprogramming fuels cell growth and proliferation. Cell Metab..

[6] Bray F., Laversanne M., Sung H., Ferlay J., Siegel R.L., Soerjomataram I., Jemal A. (2024). Global cancer statistics 2022: GLOBOCAN estimates of incidence and mortality worldwide for 36 cancers in 185 countries. CA Cancer J. Clin..

[7] Rumgay H., Ferlay J., de Martel C., Georges D., Ibrahim A.S., Zheng R., Wei W., Lemmens Valery E.P.P., Soerjomataram I. (2022). Global, regional and national burden of primary liver cancer by subtype. Eur. J. Cancer.

[8] McGlynn K.A., Petrick J.L., El-Serag H.B. (2021). Epidemiology of Hepatocellular Carcinoma. Hepatology.

[9] Stine J.G., Wentworth B.J., Zimmet A., Rinella M.E., Loomba R., Caldwell S.H., Argo C.K. (2018). Systematic review with meta-analysis: Risk of hepatocellular carcinoma in non-alcoholic steatohepatitis without cirrhosis compared to other liver diseases. Aliment. Pharmacol. Ther..

[10] Mittal S., El-Serag H.B., Sada Y.H., Kanwal F., Duan Z., Temple S., May S.B., Kramer J.R., Richardson P.A., Davila J.A. (2016). Hepatocellular Carcinoma in the Absence of Cirrhosis in US Veterans is Associated with Non-Alcoholic Fatty Liver Disease. Clin. Gastroenterol. Hepatol..

[11] Hanahan D., Weinberg R.A. (2011). Hallmarks of cancer: The next generation. Cell.

[12] Warburg O., Wind F., Negelein E. (1927). The metabolism of tumors in the body. J. Gen. Physiol..

[13] Schicht G., Seidemann L., Haensel R., Seehofer D., Damm G. (2022). Critical Investigation of the Usability of Hepatoma Cell Lines HepG2 and Huh7 as Models for the Metabolic Representation of Resectable Hepatocellular Carcinoma. Cancers.

[14] Zhu J., Thompson C.B. (2019). Metabolic regulation of cell growth and proliferation. Nat. Rev. Mol. Cell Biol..

[15] Ma X., Huang T., Chen X., Li Q., Liao M., Fu L., Huang J., Yuan K., Wang Z., Zeng Y. (2025). Molecular mechanisms in liver repair and regeneration: From physiology to therapeutics. Signal Transduct. Target. Ther..

[16] Burkard A., Dähn C., Heinz S., Zutavern A., Sonntag-Buck V., Maltman D., Przyborski S., Hewitt N.J., Braspenning J. (2012). Generation of proliferating human hepatocytes using Upcyte® technology: Characterisation and applications in induction and cytotoxicity assays. Xenobiotica.

[17] Braspenning J., Holder S., Kuepper H. (2009). Propagation of Primary Cells and Use Therof. Patent.

[18] Herzog N., Hansen M., Miethbauer S., Schmidtke K.-U., Anderer U., Lupp A., Sperling S., Seehofer D., Damm G., Scheibner K. (2016). Primary-like human hepatocytes genetically engineered to obtain proliferation competence display hepatic differentiation characteristics in monolayer and organotypical spheroid cultures. Cell Biol. Int..

[19] Scheffschick A., Babel J., Sperling S., Nerusch J., Herzog N., Seehofer D., Damm G. (2022). Primary-like Human Hepatocytes Genetically Engineered to Obtain Proliferation Competence as a Capable Application for Energy Metabolism Experiments in In Vitro Oncologic Liver Models. Biology.

[20] Matschinsky F.M. (1990). Glucokinase as glucose sensor and metabolic signal generator in pancreatic beta-cells and hepatocytes. Diabetes.

[21] Perrin-Cocon L., Vidalain P.-O., Jacquemin C., Aublin-Gex A., Olmstead K., Panthu B., Rautureau G.J.P., André P., Nyczka P., Hütt M.-T. (2021). A hexokinase isoenzyme switch in human liver cancer cells promotes lipogenesis and enhances innate immunity. Commun. Biol..

[22] Klimek F., Bannasch P. (1993). Isoenzyme shift from glucokinase to hexokinase is not an early but a late event in hepatocarcinogenesis. Carcinogenesis.

[23] DeWaal D., Nogueira V., Terry A.R., Patra K.C., Jeon S.-M., Guzman G., Au J., Long C.P., Antoniewicz M.R., Hay N. (2018). Hexokinase-2 depletion inhibits glycolysis and induces oxidative phosphorylation in hepatocellular carcinoma and sensitizes to metformin. Nat. Commun..

[24] Gong L., Cui Z., Chen P., Han H., Peng J., Leng X. (2012). Reduced survival of patients with hepatocellular carcinoma expressing hexokinase II. Med. Oncol..

[25] Grobholz R., Hacker H.J., Thorens B., Bannasch P. (1993). Reduction in the expression of glucose transporter protein GLUT 2 in preneoplastic and neoplastic hepatic lesions and reexpression of GLUT 1 in late stages of hepatocarcinogenesis. Cancer Res..

[26] Amann T., Maegdefrau U., Hartmann A., Agaimy A., Marienhagen J., Weiss T.S., Stoeltzing O., Warnecke C., Schölmerich J., Oefner P.J. (2009). GLUT1 expression is increased in hepatocellular carcinoma and promotes tumorigenesis. Am. J. Pathol..

[27] Hacker H.J., Steinberg P., Bannasch P. (1998). Pyruvate kinase isoenzyme shift from L-type to M2-type is a late event in hepatocarcinogenesis induced in rats by a choline-deficient/DL-ethionine-supplemented diet. Carcinogenesis.

[28] Mazurek S., Boschek C.B., Hugo F., Eigenbrodt E. (2005). Pyruvate kinase type M2 and its role in tumor growth and spreading. Semin. Cancer Biol..

[29] Yang W., Lu Z. (2013). Nuclear PKM2 regulates the Warburg effect. Cell Cycle.

[30] Luo W., Hu H., Chang R., Zhong J., Knabel M., O’Meally R., Cole R.N., Pandey A., Semenza G.L. (2011). Pyruvate kinase M2 is a PHD3-stimulated coactivator for hypoxia-inducible factor 1. Cell.

[31] Kim J., Zeller K.I., Wang Y., Jegga A.G., Aronow B.J., O’Donnell K.A., Dang C.V. (2004). Evaluation of myc E-box phylogenetic footprints in glycolytic genes by chromatin immunoprecipitation assays. Mol. Cell. Biol..

[32] Osthus R.C., Shim H., Kim S., Li Q., Reddy R., Mukherjee M., Xu Y., Wonsey D., Lee L.A., Dang C.V. (2000). Deregulation of glucose transporter 1 and glycolytic gene expression by c-Myc. J. Biol. Chem..

[33] Huang L.E. (2008). Carrot and stick: HIF-alpha engages c-Myc in hypoxic adaptation. Cell Death Differ..

[34] Kim J., Gao P., Liu Y.-C., Semenza G.L., Dang C.V. (2007). Hypoxia-inducible factor 1 and dysregulated c-Myc cooperatively induce vascular endothelial growth factor and metabolic switches hexokinase 2 and pyruvate dehydrogenase kinase 1. Mol. Cell. Biol..

[35] Dang C.V., Kim J., Gao P., Yustein J. (2008). The interplay between MYC and HIF in cancer. Nat. Rev. Cancer.

[36] Huang D., Li T., Wang L., Zhang L., Yan R., Li K., Xing S., Wu G., Hu L., Jia W. (2016). Hepatocellular carcinoma redirects to ketolysis for progression under nutrition deprivation stress. Cell Res..

[37] Martinez-Outschoorn U.E., Lin Z., Whitaker-Menezes D., Howell A., Sotgia F., Lisanti M.P. (2012). Ketone body utilization drives tumor growth and metastasis. Cell Cycle.

[38] WHO Classification of Tumours Editorial Board (2019). Digestive System Tumours.

[39] Pfeiffer E., Kegel V., Zeilinger K., Hengstler J.G., Nüssler A.K., Seehofer D., Damm G. (2015). Featured Article: Isolation, characterization, and cultivation of human hepatocytes and non-parenchymal liver cells. Exp. Biol. Med..

[40] Kegel V., Deharde D., Pfeiffer E., Zeilinger K., Seehofer D., Damm G. (2016). Protocol for Isolation of Primary Human Hepatocytes and Corresponding Major Populations of Non-parenchymal Liver Cells. J. Vis. Exp..

[41] Damm G., Schicht G., Zimmermann A., Rennert C., Fischer N., Kießig M., Wagner T., Kegel V., Seehofer D. (2019). Effect of glucose and insulin supplementation on the isolation of primary human hepatocytes. EXCLI J..

[42] Bustin S.A., Benes V., Garson J.A., Hellemans J., Huggett J., Kubista M., Mueller R., Nolan T., Pfaffl M.W., Shipley G.L. (2009). The MIQE guidelines: Minimum information for publication of quantitative real-time PCR experiments. Clin. Chem..

[43] Taylor S.C., Nadeau K., Abbasi M., Lachance C., Nguyen M., Fenrich J. (2019). The Ultimate qPCR Experiment: Producing Publication Quality, Reproducible Data the First Time. Trends Biotechnol..

[44] Lin X., Xiao Z., Chen T., Liang S.H., Guo H. (2020). Glucose Metabolism on Tumor Plasticity, Diagnosis, and Treatment. Front. Oncol..

[45] Roach P.J. (2002). Glycogen and its metabolism. Curr. Mol. Med..

[46] Khan T., Sullivan M.A., Gunter J.H., Kryza T., Lyons N., He Y., Hooper J.D. (2020). Revisiting Glycogen in Cancer: A Conspicuous and Targetable Enabler of Malignant Transformation. Front. Oncol..

[47] Lust W.D., Passonneau J.V., Crites S.K. (1975). The measurement of glycogen in tissues by amylo-alpha-1,4-alpha-1,6-glucosidase after the destruction of preexisting glucose. Anal. Biochem..

[48] Pilling J., Garside H., Ainscow E. (2010). Development of a quantitative 96-well method to image glycogen storage in primary rat hepatocytes. Mol. Cell. Biochem..

[49] Katz J., Golden S., Wals P.A. (1976). Stimulation of hepatic glycogen synthesis by amino acids. Proc. Natl. Acad. Sci. USA.

[50] Cascio S., Zaret K.S. (1991). Hepatocyte differentiation initiates during endodermal-mesenchymal interactions prior to liver formation. Development.

[51] Sun L., Zhang H., Gao P. (2022). Metabolic reprogramming and epigenetic modifications on the path to cancer. Protein Cell.

[52] Lunt S.Y., Vander Heiden M.G. (2011). Aerobic glycolysis: Meeting the metabolic requirements of cell proliferation. Annu. Rev. Cell Dev. Biol..

[53] Pavlova N.N., Thompson C.B. (2016). The Emerging Hallmarks of Cancer Metabolism. Cell Metab..

[54] Li J., Ning G., Duncan S.A. (2000). Mammalian hepatocyte differentiation requires the transcription factor HNF-4alpha. Genes Dev..

[55] Nekvindova J., Mrkvicova A., Zubanova V., Hyrslova Vaculova A., Anzenbacher P., Soucek P., Radova L., Slaby O., Kiss I., Vondracek J. (2020). Hepatocellular carcinoma: Gene expression profiling and regulation of xenobiotic-metabolizing cytochromes P450. Biochem. Pharmacol..

[56] Faubert B., Solmonson A., DeBerardinis R.J. (2020). Metabolic reprogramming and cancer progression. Science.

[57] Saggese P., Sellitto A., Martinez C.A., Giurato G., Nassa G., Rizzo F., Tarallo R., Scafoglio C. (2020). Metabolic Regulation of Epigenetic Modifications and Cell Differentiation in Cancer. Cancers.

[58] Hass H.G., Jobst J., Scheulen M., Vogel U., Nehls O. (2015). Gene expression analysis for evaluation of potential biomarkers in hepatocellular carcinoma. Anticancer Res..

[59] Nakatsura T., Yoshitake Y., Senju S., Monji M., Komori H., Motomura Y., Hosaka S., Beppu T., Ishiko T., Kamohara H. (2003). Glypican-3, overexpressed specifically in human hepatocellular carcinoma, is a novel tumor marker. Biochem. Biophys. Res. Commun..

[60] Chang C., Zhao W., Luo Y., Xi L., Chen S., Zhao C., Wang G., Guo J., Xu C. (2017). Serine peptidase inhibitor Kazal type I (SPINK1) promotes BRL-3A cell proliferation via p38, ERK, and JNK pathways. Cell Biochem. Funct..

[61] Huang K., Xie W., Wang S., Li Q., Wei X., Chen B., Hua Y., Li S., Peng B., Shen S. (2021). High SPINK1 Expression Predicts Poor Prognosis and Promotes Cell Proliferation and Metastasis of Hepatocellular Carcinoma. J. Investig. Surg..

[62] Gao C.-L., Wang G.-W., Yang G.-Q., Yang H., Zhuang L. (2018). Karyopherin subunit-α 2 expression accelerates cell cycle progression by upregulating CCNB2 and CDK1 in hepatocellular carcinoma. Oncol. Lett..

[63] Guo X., Wang Z., Zhang J., Xu Q., Hou G., Yang Y., Dong C., Liu G., Liang C., Liu L. (2019). Upregulated KPNA2 promotes hepatocellular carcinoma progression and indicates prognostic significance across human cancer types. Acta Biochim. Biophys. Sin..

[64] Lee C.-W., Tsai H.-I., Lee W.-C., Huang S.-W., Lin C.-Y., Hsieh Y.-C., Kuo T., Chen C.-W., Yu M.-C. (2019). Normal Alpha-Fetoprotein Hepatocellular Carcinoma: Are They Really Normal?. J. Clin. Med..

[65] Vargas-López M., Quiroz-Vicente C.A., Pérez-Hernández N., Gómez-Chávez F., Bañuelos-Hernández A.E., Pérez-Hernández E. (2024). The ketone body β-Hydroxybutyrate as a fuel source of chondrosarcoma cells. Heliyon.

[66] Liu Z., Li Y., Liu Y., Yang D., Jiao Y., Liu Y. (2021). Expression and clinical significance of BDH1 in liver cancer. Medicine.

[67] Luo W., Wu S., Zhang F., Chen X., Ma Y., Mo Y. (2022). Decreased expression of 3-hydroxybutyrate dehydrogenase 1 is a prognostic marker and promotes tumor progression in hepatocellular carcinoma. Pathol. Res. Pract..

[68] Barajas J.M., Reyes R., Guerrero M.J., Jacob S.T., Motiwala T., Ghoshal K. (2018). The role of miR-122 in the dysregulation of glucose-6-phosphate dehydrogenase (G6PD) expression in hepatocellular cancer. Sci. Rep..

[69] Jiang S., Zhang L.-F., Zhang H.-W., Hu S., Lu M.-H., Liang S., Li B., Li Y., Li D., Wang E.-D. (2012). A novel miR-155/miR-143 cascade controls glycolysis by regulating hexokinase 2 in breast cancer cells. EMBO J..

[70] Kaposi-Novak P., Libbrecht L., Woo H.G., Lee Y.-H., Sears N.C., Coulouarn C., Conner E.A., Factor V.M., Roskams T., Thorgeirsson S.S. (2009). Central role of c-Myc during malignant conversion in human hepatocarcinogenesis. Cancer Res..

[71] Sequera C., Grattarola M., Holczbauer A., Dono R., Pizzimenti S., Barrera G., Wangensteen K.J., Maina F. (2022). MYC and MET cooperatively drive hepatocellular carcinoma with distinct molecular traits and vulnerabilities. Cell Death Dis..

[72] Min Z., Xunlei Z., Haizhen C., Wenjing Z., Haiyan Y., Xiaoyun L., Jianyun Z., Xudong C., Aiguo S. (2021). The Clinicopathologic and Prognostic Significance of c-Myc Expression in Hepatocellular Carcinoma: A Meta-Analysis. Front. Bioinform..

[73] Sumi T., Tsuneyoshi N., Nakatsuji N., Suemori H. (2007). Apoptosis and differentiation of human embryonic stem cells induced by sustained activation of c-Myc. Oncogene.

[74] Cliff T.S., Wu T., Boward B.R., Yin A., Yin H., Glushka J.N., Prestegaard J.H., Dalton S. (2017). MYC Controls Human Pluripotent Stem Cell Fate Decisions through Regulation of Metabolic Flux. Cell Stem Cell.

[75] Li L., Jin R., Zhang X., Lv F., Liu L., Liu D., Liu K., Li N., Chen D. (2012). Oncogenic activation of glypican-3 by c-Myc in human hepatocellular carcinoma. Hepatology.

[76] Mitchell G.A., Kassovska-Bratinova S., Boukaftane Y., Robert M.F., Wang S.P., Ashmarina L., Lambert M., Lapierre P., Potier E. (1995). Medical aspects of ketone body metabolism. Clin. Investig. Med..

[77] Fukao T., Song X.Q., Mitchell G.A., Yamaguchi S., Sukegawa K., Orii T., Kondo N. (1997). Enzymes of ketone body utilization in human tissues: Protein and messenger RNA levels of succinyl-coenzyme A (CoA):3-ketoacid CoA transferase and mitochondrial and cytosolic acetoacetyl-CoA thiolases. Pediatr. Res..

[78] Zhang S., Xie C. (2017). The role of OXCT1 in the pathogenesis of cancer as a rate-limiting enzyme of ketone body metabolism. Life Sci..

[79] Stagg D.B., Gillingham J.R., Nelson A.B., Lengfeld J.E., d’Avignon D.A., Puchalska P., Crawford P.A. (2021). Diminished ketone interconversion, hepatic TCA cycle flux, and glucose production in D-β-hydroxybutyrate dehydrogenase hepatocyte-deficient mice. Mol. Metab..

[80] Puchalska P., Crawford P.A. (2017). Multi-dimensional Roles of Ketone Bodies in Fuel Metabolism, Signaling, and Therapeutics. Cell Metab..

[81] Williamson D.H., Bates M.W., Page M.A., Krebs H.A. (1971). Activities of enzymes involved in acetoacetate utilization in adult mammalian tissues. Biochem. J..

[82] Owen O.E., Reichard G.A. (1971). Human forearm metabolism during progressive starvation. J. Clin. Investig..

[83] Fukao T., Mitchell G., Sass J.O., Hori T., Orii K., Aoyama Y. (2014). Ketone body metabolism and its defects. J. Inherit. Metab. Dis..

[84] Kammerer S., Küpper J.-H. (2018). Human hepatocyte systems for in vitro toxicology analysis. J. Cell. Biotechnol..

