# Supplementary material for: Investigation and Distinction of Energy Metabolism in Proliferating Hepatocytes and Hepatocellular Carcinoma Cells
Source: Cells. 2025 Aug 14;14(16):1254. doi: 10.3390/cells14161254 (PMC12384642; doi:10.3390/cells14161254)
Supplement: Supplementary file 1 [file cells-14-01254-s001.zip › cells-3737592-supplementary.pdf]

cells MDPI

*Supplementary data*

# Investigation and Distinction of Energy Metabolism in Proliferating Hepatocytes and Hepatocellular Carcinoma Cells

**Julia Nerusch [1,2,†], Gerda Schicht [1,2,†], Natalie Herzog [3], Jan-Heiner Küpper [3], Daniel Seehofer [1,2] and Georg Damm [1,2,*]**

1 Department of Hepatobiliary Surgery and Visceral Transplantation, University Hospital, Leipzig University, 04103 Leipzig, Germany; julia.nerusch@medizin.uni-leipzig.de (J.N.); gerda.schicht@sikt.uni-leipzig.de (G.S.); daniel.seehofer@medizin.uni-leipzig.de (D.S.)
2 Saxonian Incubator for Clinical Translation (SIKT), Leipzig University, 04103 Leipzig, Germany
3 Faculty of Science, Brandenburg University of Technology Cottbus-Senftenberg, 01968 Senftenberg, Germany; herzog@b-tu.de (N.H.); jan-heiner.kuepper@b-tu.de (J.-H.K.)
* Correspondence: georg.damm@medizin.uni-leipzig.de
† These authors contributed equally to this work.

**Table S1.** Gene specific primers purchased from Qiagen (Hilden, Germany) for RT-qPCR analyses.

| Article | Gene name | Article number |
|---|---|---|
| Hs_BDH1_1_SG QuantiTect® Primer Assay | *BDH1* | QT01673525 |
| Hs_CDH1_1_SG QuantiTect Primer Assay | *E-Cadherin* | QT00080143 |
| Hs_CYP3A4_1_SG QuantiTect Primer Assay | *CYP3A4* | QT00067396 |
| Hs_FOXO1_1_SG QuantiTect® Primer Assay | *FOXO1* | QT00044247 |
| Hs_GAPDH_vb.1_SG QuantiTect® Primer Assay | *GAPDH* | QT02504278 |
| Hs_GCK_1_SG QuantiTect Primer Assay | *GCK/HK4* | QT00000812 |
| Hs_GSK3A_1_SG QuantiTect® Primer Assay | *GSK3A* | QT00075306 |
| Hs_GSK3B_1_SG QuantiTect® Primer Assay | *GSK3B* | QT00057134 |
| Hs_GUSB_1_SG QuantiTect® Primer Assay | *GUSB* | QT00046046 |
| Hs_HIF1A_1_SG QuantiTect® Primer Assay | *HIF1A* | QT00083664 |
| Hs_HK1_1_SG QuantiTect Primer Assay | *HK1* | QT00045514 |
| Hs_HK2_1_SG QuantiTect Primer Assay | *HK2* | QT00013209 |
| Hs_HMGCL_1_SG QuantiTect® Primer Assay | *HMGCL* | QT00088921 |
| Hs_HNF4A_1_SG QuantiTect Primer Assay | *HNF4A* | QT00019411 |
| Hs_LDHA_1_SG QuantiTect® Primer Assay | *LDHA* | QT00001687 |
| Hs_MYC_1_SG QuantiTect Primer Assay | *c-MYC* | QT00035406 |
| Hs_PKLR_1_SG QuantiTect Primer Assay | *PKL* | QT00016156 |
| Hs_PKM_1_SG QuantiTect Primer Assay | *PKM* | QT00028875 |
| Hs_RRN18S_1_SG QuantiTect Primer Assay | *RRN18S* | QT00199367 |
| Hs_SLC2A1_1_SG QuantiTect Primer Assay | *GLUT1* | QT00068957 |
| Hs_SLC2A2_2_SG QuantiTect Primer Assay | *GLUT2* | QT01008399 |

Primers were solved in 1.1 ml TE Buffer.

 https://doi.org/10.3390/cells14161254

**Table S2.** Primary antibodies used for Western blot analyses.

| Target | article | company | Article number | Protein concentration [µg] | Positive control | Gel concentration [%] |
|---|---|---|---|---|---|---|
| BDH1 | BDH1 Monoclonal Antibody (1A5) | Thermo Fisher Scientific, Waltham, US | MA5-15594 | 20 | kidney (mouse) | 15 |
| c-MYC | c-Myc (D84C12) Rabbit mAb | Cell Signaling Technology, Masachusetts, US | 5605S | 25 | Brain (mouse) | 12 |
| FOXO1 | Fox01 (**L**27) Rabbit Ab | Cell Signaling Technology, Masachusetts, US | 9454S | 30 | heart (mouse) | 12 |
| GCK | Recombinant Anti-Glucokinase antibody [EPR10374] | Abcam, Cambridge, GB | ab155962 | 10 | PHH (human) | 12 |
| GLUT1 | Glut1 (D3J3A) Rabbit mAb | Cell Signaling Technology, Masachusetts, US | 12939S | 15 | Gallbladder (human) | 12 |
| GLUT2 | Recombinant Anti-Glucose Transporter GLUT2 antibody [EPR16550] | Abcam, Cambridge, GB | ab192599 | 15 | Gallbladder (human) | 12 |
| GSK3A | GSK-3α (D80E6) Rabbit mAb #4337 | Cell Signaling Technology, Masachusetts, US | 4337S | 15 | Brain (mouse) | 15 |
| GSK3B | GSK-3β (3D10) Mouse mAb #9832 | Cell Signaling Technology, Masachusetts, US | 9832S | 15 | Brain (mouse) | 15 |
| HIF1A | HIF-1α (D2**U**3T) Rabbit mAb | Cell Signaling Technology, Masachusetts, US | 14179S | 25 | HepG2 in hypoxie | 8 |
| HK1 | Hexokinase I (C35C4) Rabbit mAb | Cell Signaling Technology, Masachusetts, US | 2024S | 20 | lung (mouse) | 12 |
| HK2 | Hexokinase II (C64G5) Rabbit mAb | Cell Signaling Technology, Masachusetts, US | 2867S | 30 | HepG2 | 12 |
| HMGCL | HMGCL Polyclonal Antibody | Thermo Fisher Scientific, Waltham, US | PA5-21996 | 30 | Lung (mouse) | 15 |
| LDHA | LDHA (C4B5) Rabbit mAb #3582 | Cell Signaling Technology, Masachusetts, US | 3582S | 25 | THP-1 | 15 |
| PKL | Recombinant Anti-PKLR antibody [EPR11093] | Abcam, Cambridge, GB | ab171744 | 10 | PHH (human) | 12 |
| PKM | PKM1/2 (C103A3) Rabbit mAb | Cell Signaling Technology, Masachusetts, US | 3190S | 15 | Gallbladder (human) | 15 |

All antibodies were diluted 1:1,000 in Intercept Blocking Buffer with 0.1 % TWEEN except from HMGCL 1:3,000.

**Table S3.** Secondary antibodies used for Western blot analyses.

| article | company | Article number |
|---|---|---|

| | | |
|---|---|---|
| IRDye® 800CW Goat anti-Mouse IgG Secondary Antibody | Li-Cor Biosciences, Lincoln, US | 926-32210 |
| IRDye® 800CW Goat anti-Rabbit IgG Secondary Antibody | Li-Cor Biosciences, Lincoln, US | 926-32211 |

Antibodies were diluted 1:10,000 in Intercept Blocking Buffer with 0.1 % Tween.

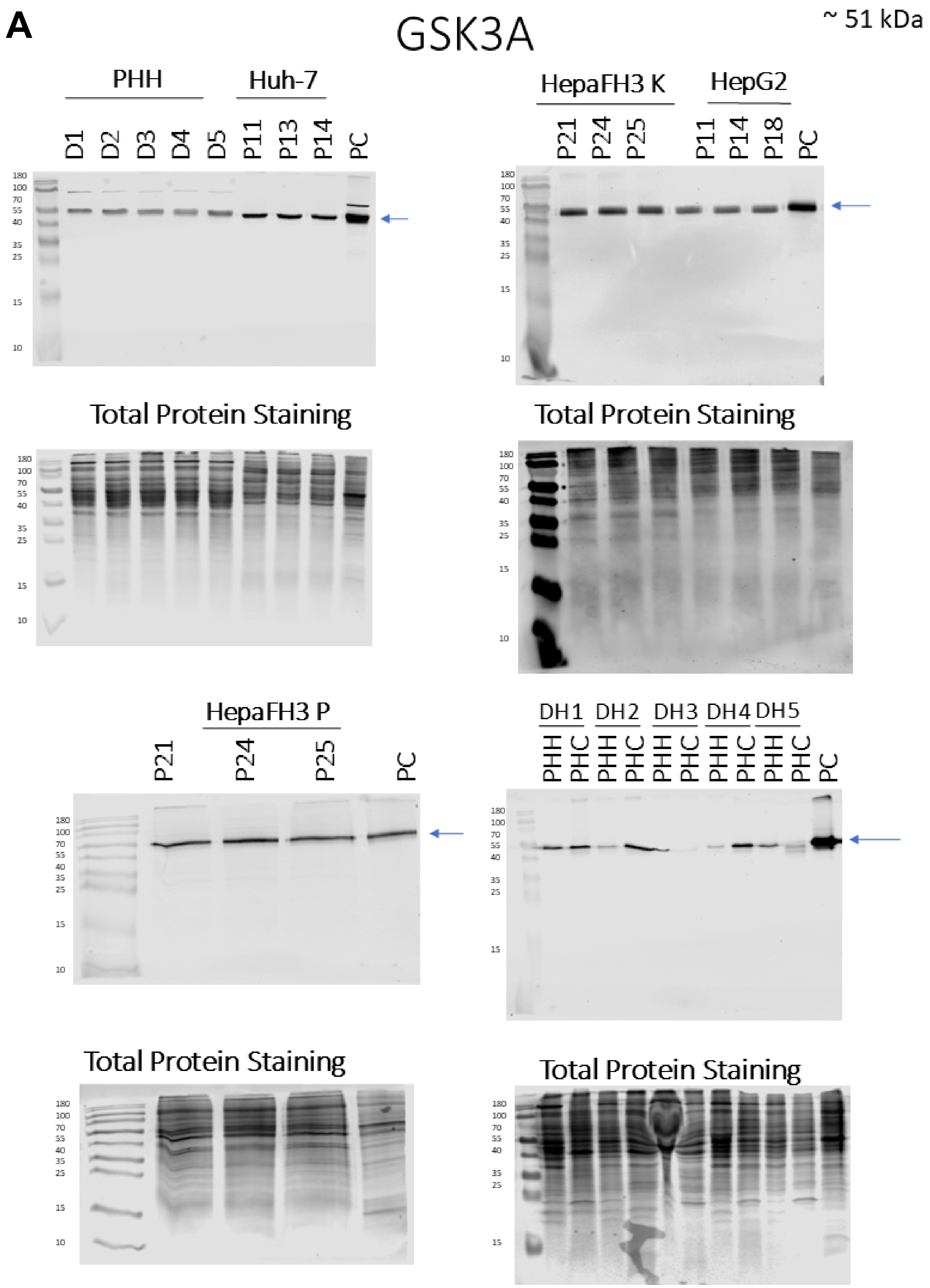
A
GSK3A
~ 51 kDa
PHH
Huh-7
D1 D2 D3 D4 D5 P11 P13 P14 PC
HepaFH3 K
HepG2
P21 P24 P25 P11 P14 P18 PC
180 100 70 55 40 35 25 15 10
Total Protein Staining
Total Protein Staining
HepaFH3 P
P21 P24 P25 PC
DH1 DH2 DH3 DH4 DH5
PHH PHC PHH PHC PHH PHC PHH PHC PHH PHC PC
Total Protein Staining
Total Protein Staining

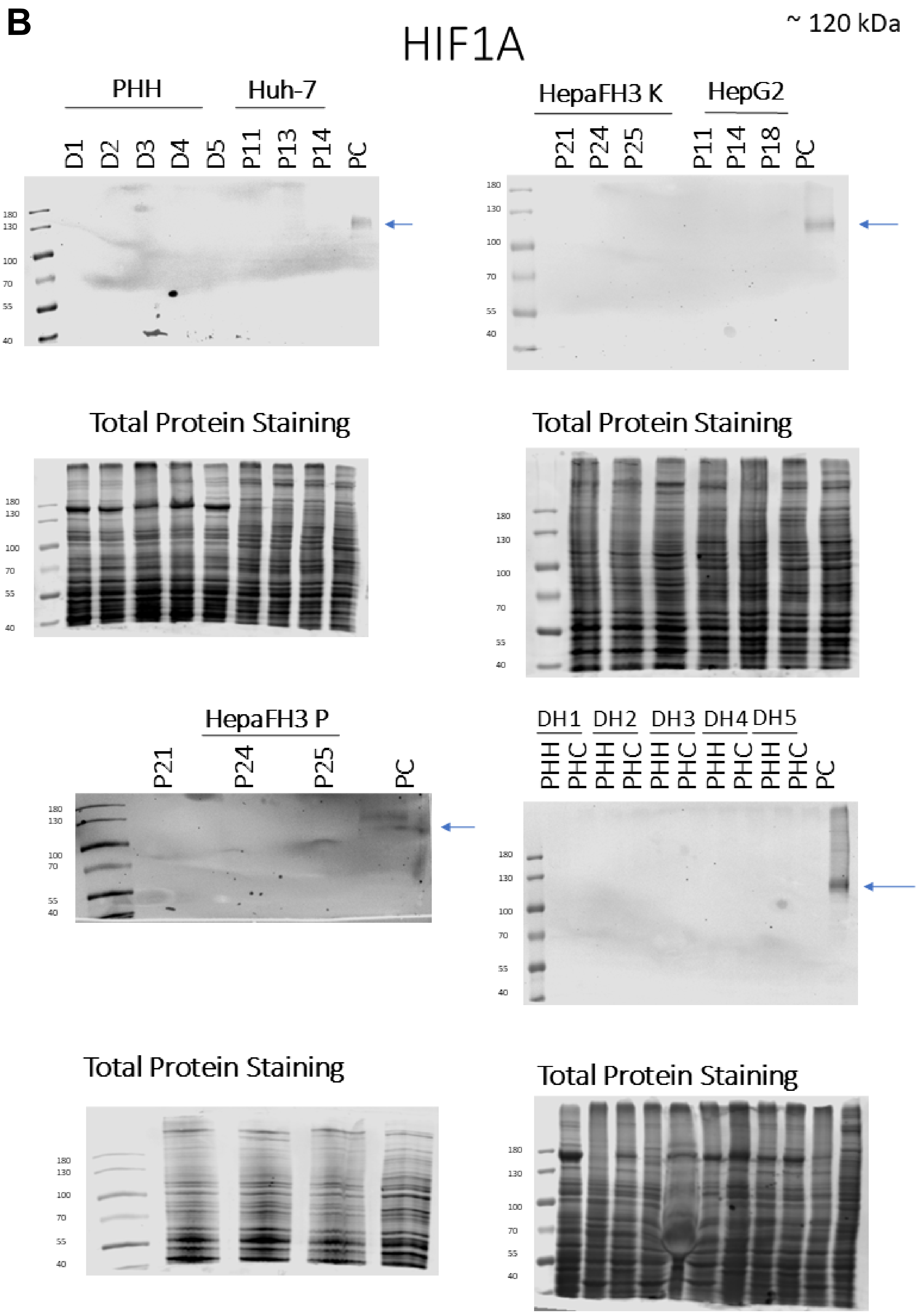
B
HIF1A
~ 120 kDa
PHH
Huh-7
D1 D2 D3 D4 D5 P11 P13 P14 PC
HepaFH3 K
HepG2
P21 P24 P25 P11 P14 P18 PC
Total Protein Staining
Total Protein Staining
HepaFH3 P
P21 P24 P25 PC
DH1 DH2 DH3 DH4 DH5
PHH PHC PHH PHC PHH PHC PHH PHC PHH PHC PC
Total Protein Staining
Total Protein Staining
180
130
100
70
55
40

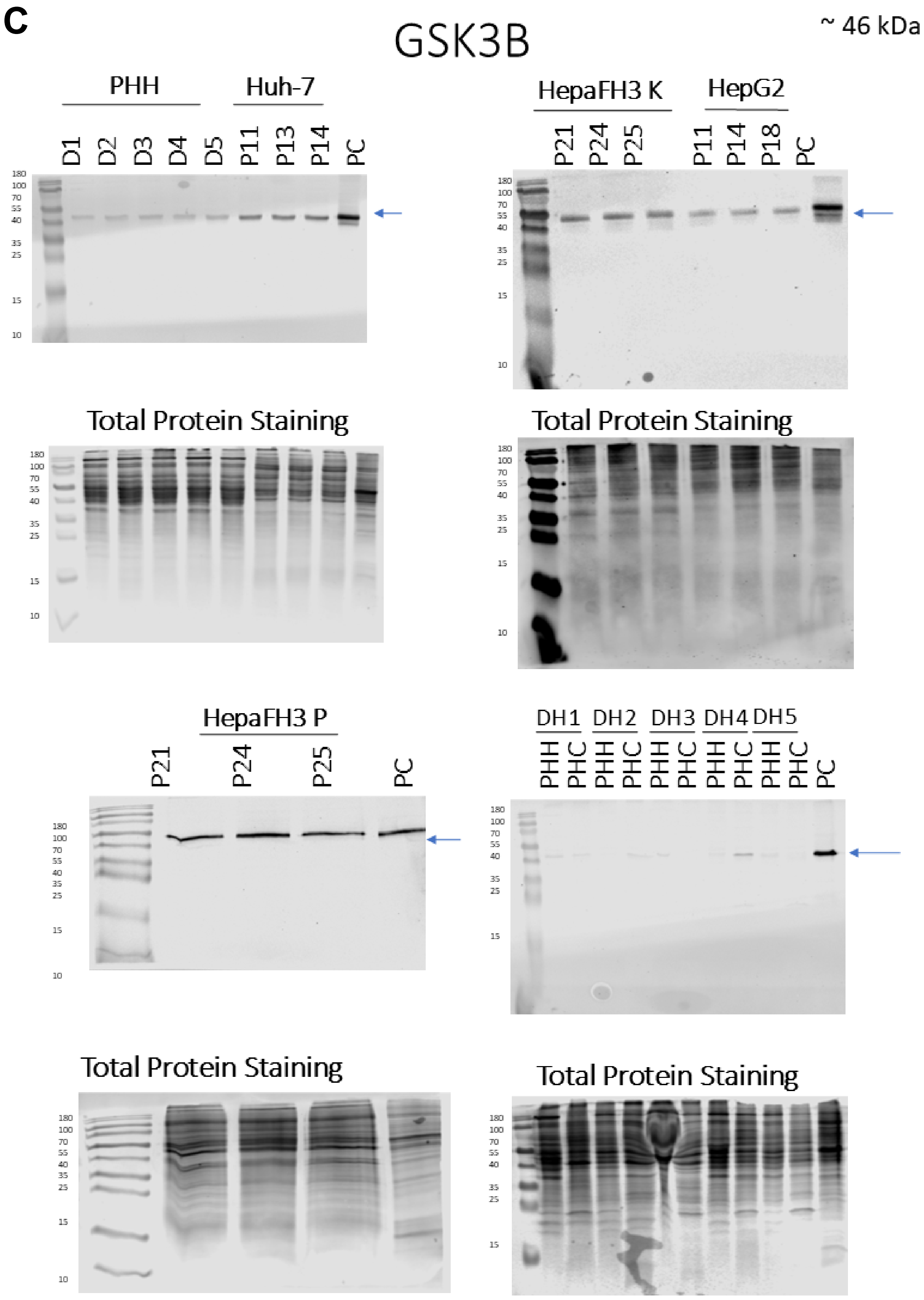
C
GSK3B
~ 46 kDa
PHH
Huh-7
D1
D2
D3
D4
D5
P11
P13
P14
PC
HepaFH3 K
HepG2
P21
P24
P25
P11
P14
P18
PC
180
100
70
55
40
35
25
15
10
Total Protein Staining
Total Protein Staining
HepaFH3 P
P21
P24
P25
PC
DH1
DH2
DH3
DH4
DH5
PHH
PHC
PHH
PHC
PHH
PHC
PHH
PHC
PHH
PHC
PC
Total Protein Staining
Total Protein Staining

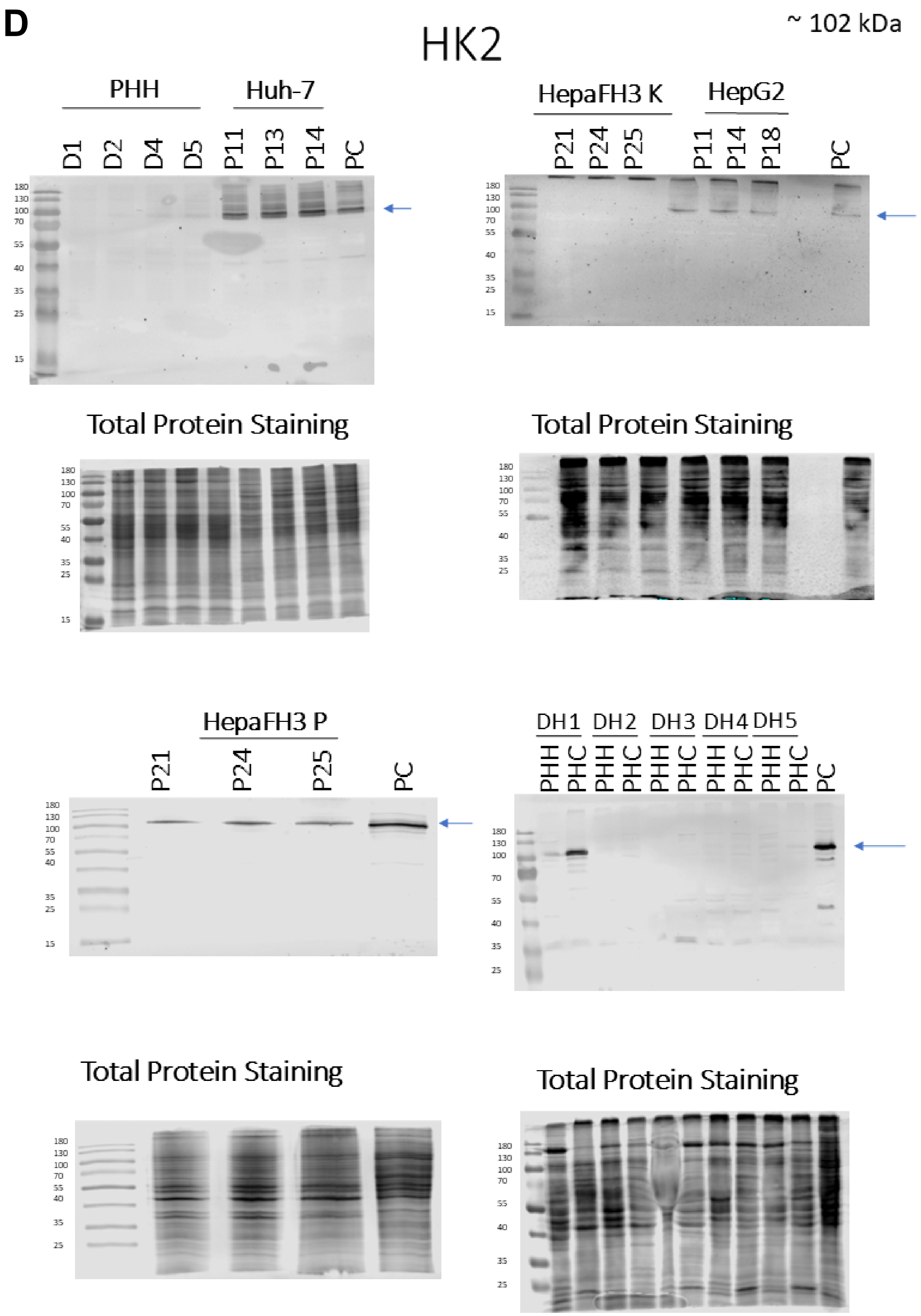
D
HK2
~ 102 kDa
PHH
Huh-7
D1 D2 D4 D5 P11 P13 P14 PC
HepaFH3 K
HepG2
P21 P24 P25 P11 P14 P18 PC
Total Protein Staining
Total Protein Staining
HepaFH3 P
P21 P24 P25 PC
DH1 DH2 DH3 DH4 DH5
PHH PHC PHH PHC PHH PHC PHH PHC PHH PHC PC
Total Protein Staining
Total Protein Staining

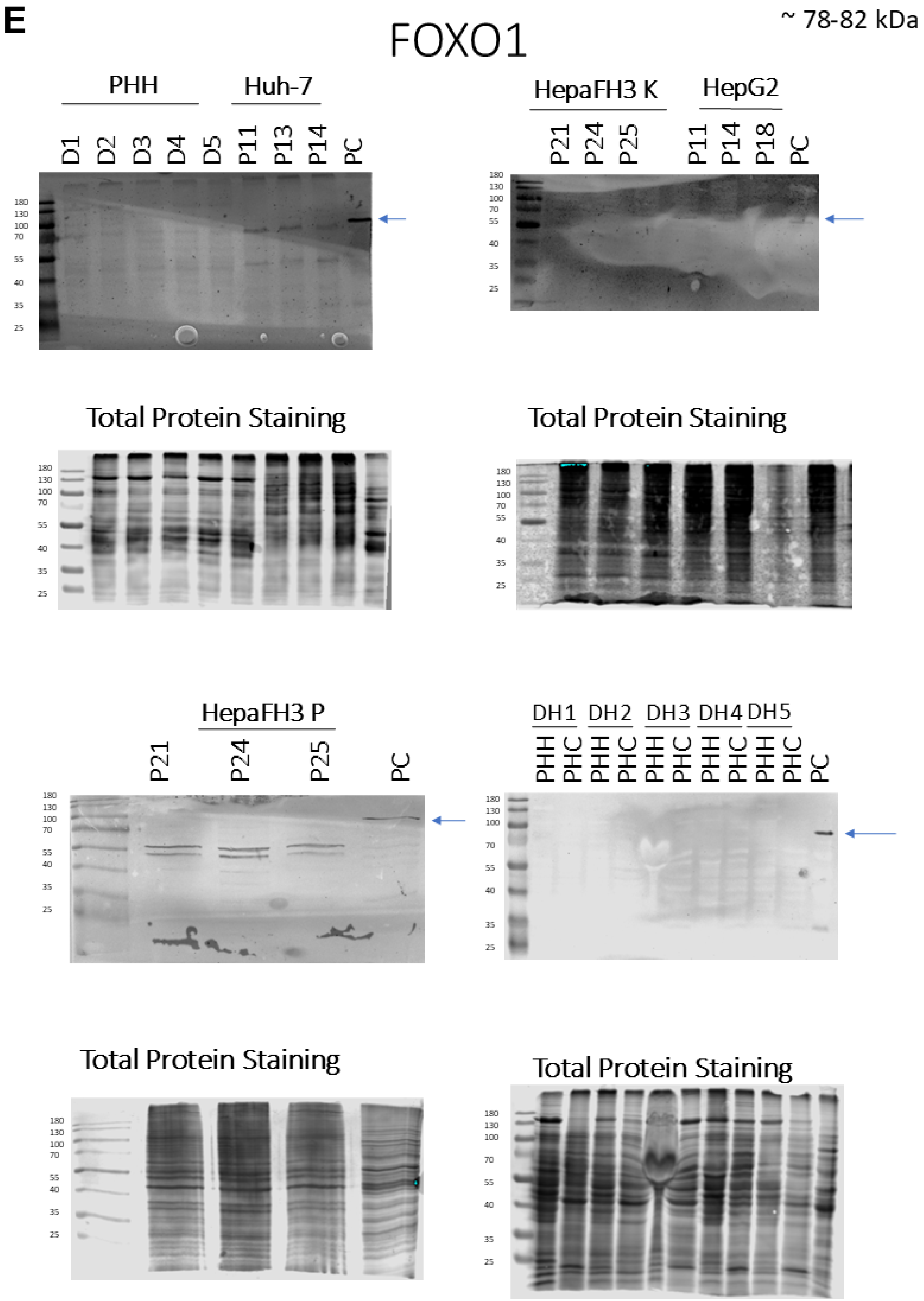
E
FOXO1
~ 78-82 kDa
PHH
Huh-7
D1
D2
D3
D4
D5
P11
P13
P14
PC
HepaFH3 K
HepG2
P21
P24
P25
P11
P14
P18
PC
180
130
100
70
55
40
35
25
Total Protein Staining
Total Protein Staining
HepaFH3 P
P21
P24
P25
PC
DH1
DH2
DH3
DH4
DH5
PHH
PHC
PHH
PHC
PHH
PHC
PHH
PHC
PHH
PHC
PC
Total Protein Staining
Total Protein Staining

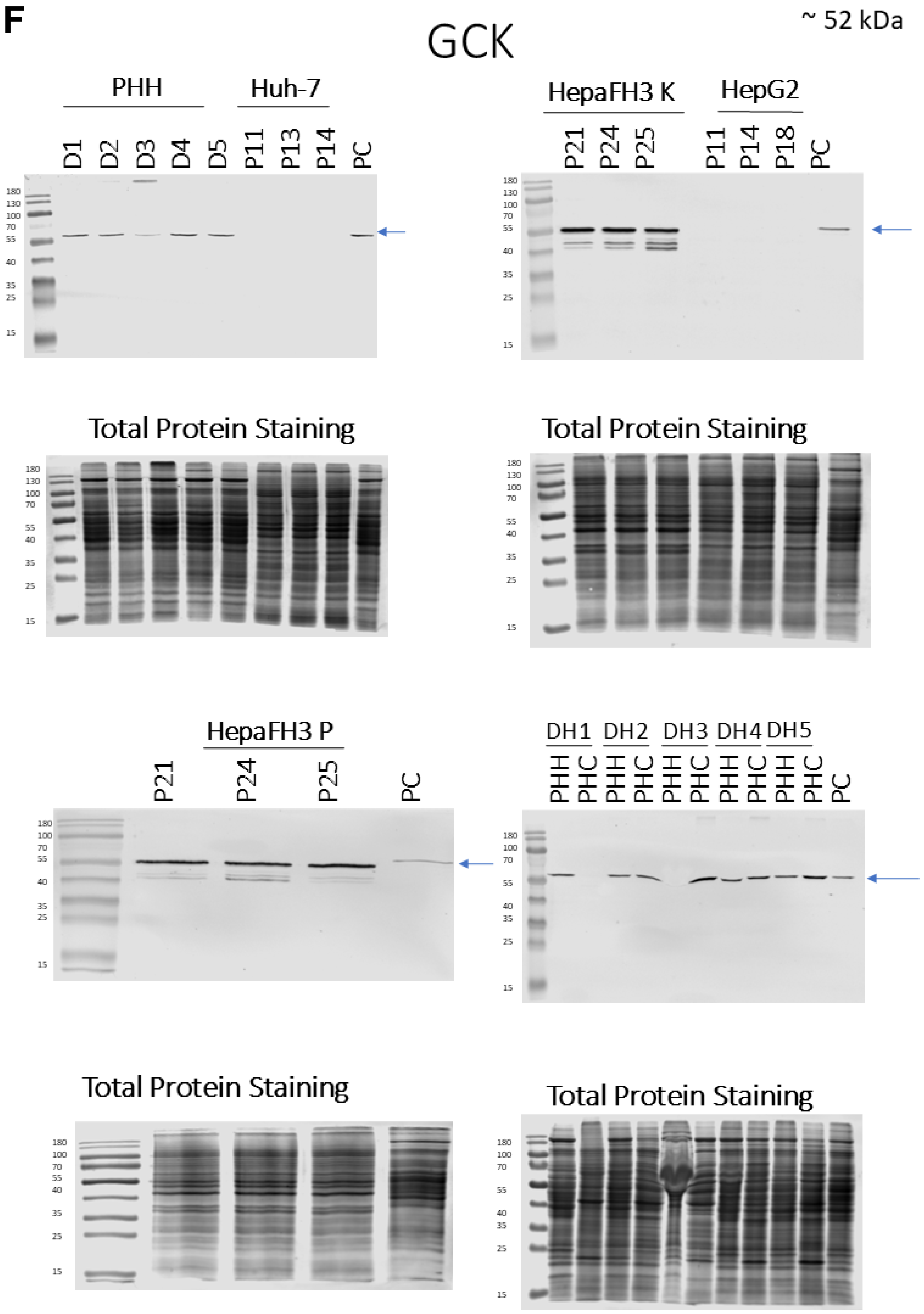
F
GCK
~ 52 kDa
PHH
Huh-7
D1 D2 D3 D4 D5 P11 P13 P14 PC
HepaFH3 K
HepG2
P21 P24 P25 P11 P14 P18 PC
Total Protein Staining
Total Protein Staining
HepaFH3 P
P21 P24 P25 PC
DH1 DH2 DH3 DH4 DH5
PHH PHC PHH PHC PHH PHC PHH PHC PHH PHC PC
Total Protein Staining
Total Protein Staining
180
130
100
70
55
40
35
25
15

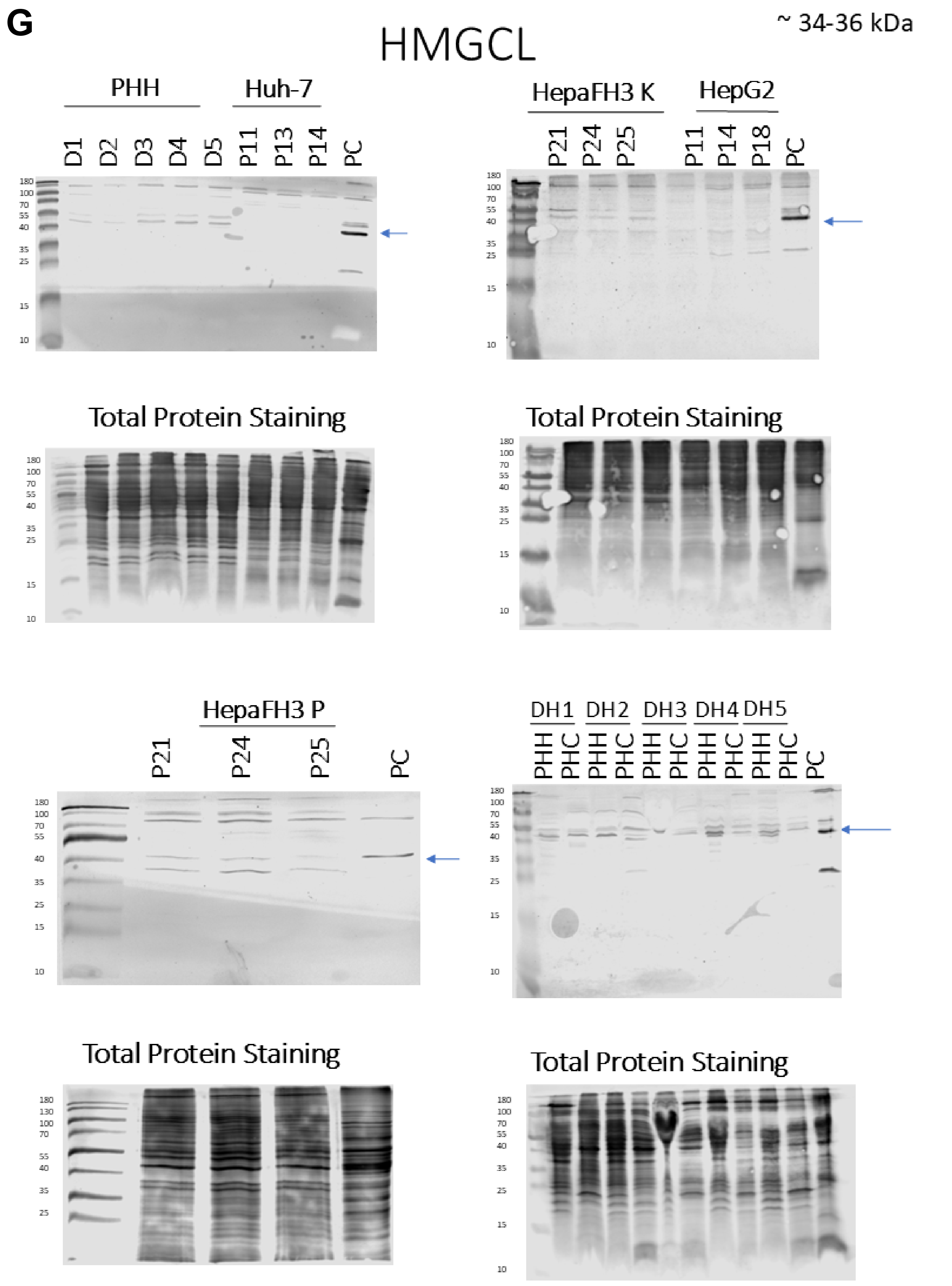
G
HMGCL
~ 34-36 kDa
PHH
Huh-7
D1 D2 D3 D4 D5 P11 P13 P14 PC
HepaFH3 K
HepG2
P21 P24 P25 P11 P14 P18 PC
Total Protein Staining
Total Protein Staining
HepaFH3 P
P21 P24 P25 PC
DH1 DH2 DH3 DH4 DH5
PHH PHC PHH PHC PHH PHC PHH PHC PHH PHC PC
Total Protein Staining
Total Protein Staining

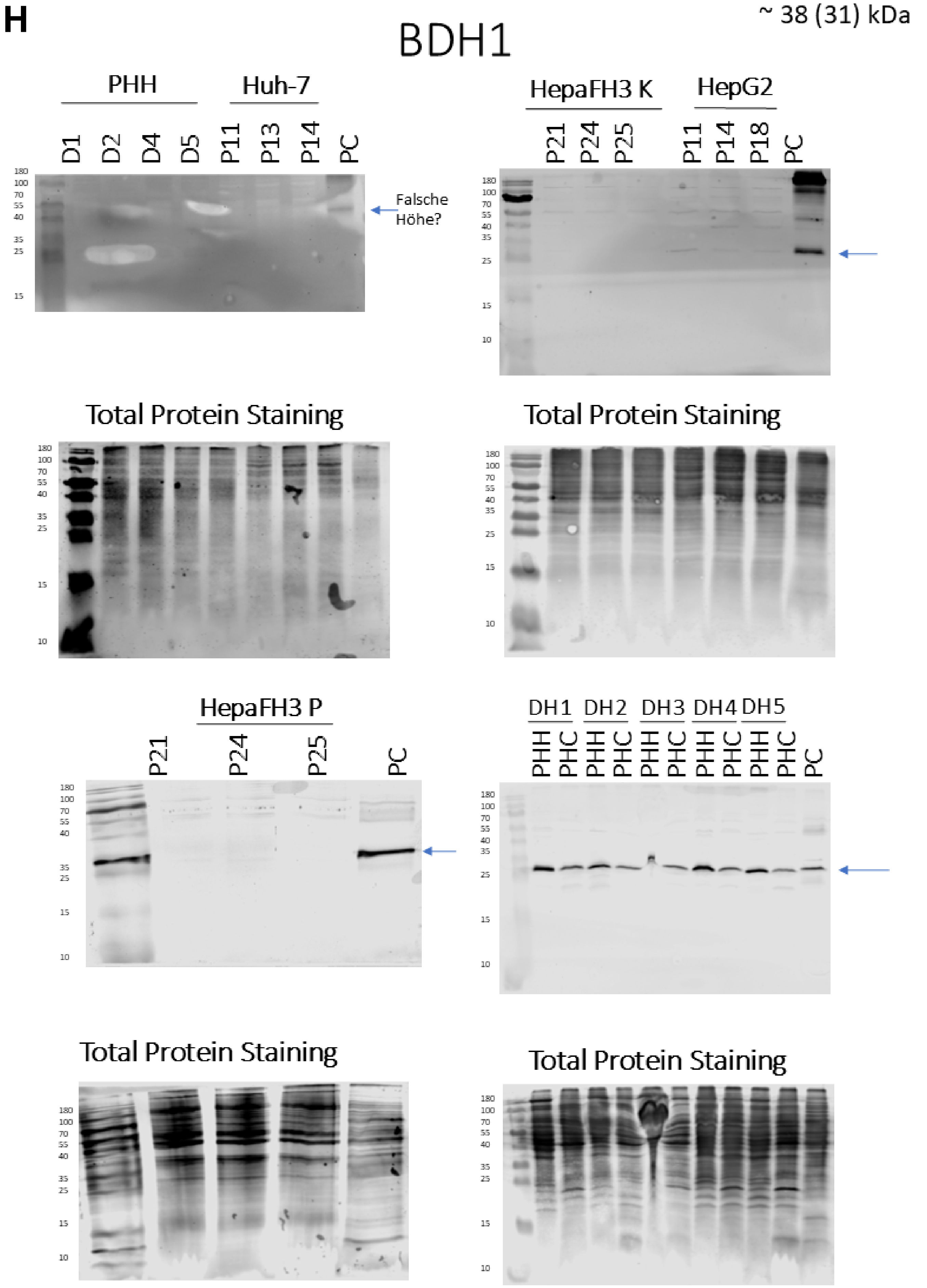
H
BDH1
~ 38 (31) kDa
PHH
Huh-7
D1
D2
D4
D5
P11
P13
P14
PC
Falsche Höhe?
HepaFH3 K
HepG2
P21
P24
P25
P11
P14
P18
PC
Total Protein Staining
Total Protein Staining
HepaFH3 P
P21
P24
P25
PC
DH1
DH2
DH3
DH4
DH5
PHH
PHC
PHH
PHC
PHH
PHC
PHH
PHC
PHH
PHC
PC
Total Protein Staining
Total Protein Staining

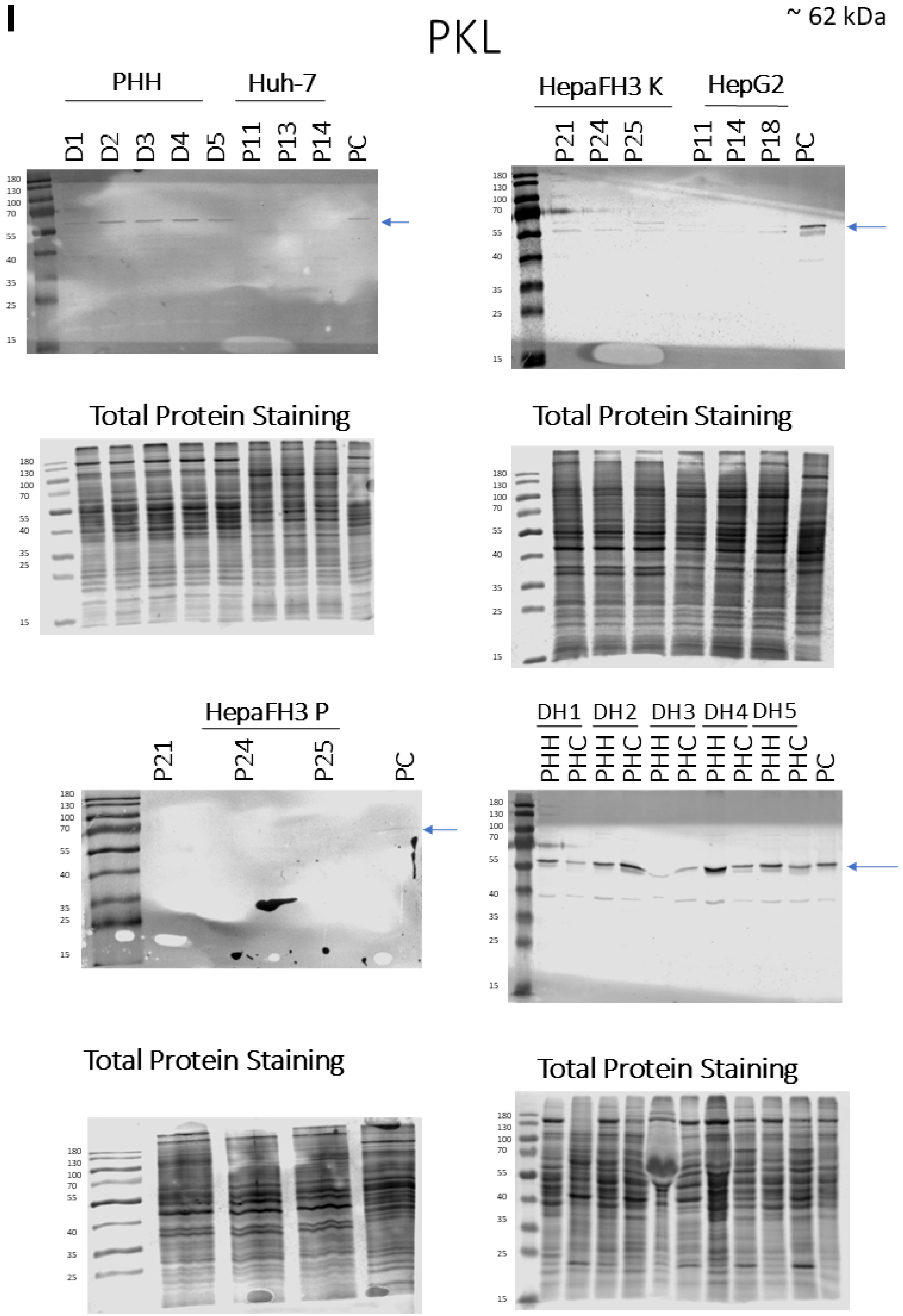
I
PKL
~ 62 kDa
PHH
Huh-7
D1 D2 D3 D4 D5 P11 P13 P14 PC
180 130 100 70 55 40 35 25 15
HepaFH3 K
HepG2
P21 P24 P25 P11 P14 P18 PC
Total Protein Staining
HepaFH3 P
P21 P24 P25 PC
DH1 DH2 DH3 DH4 DH5
PHH PHC PHH PHC PHH PHC PHH PHC PHH PHC PC
Total Protein Staining

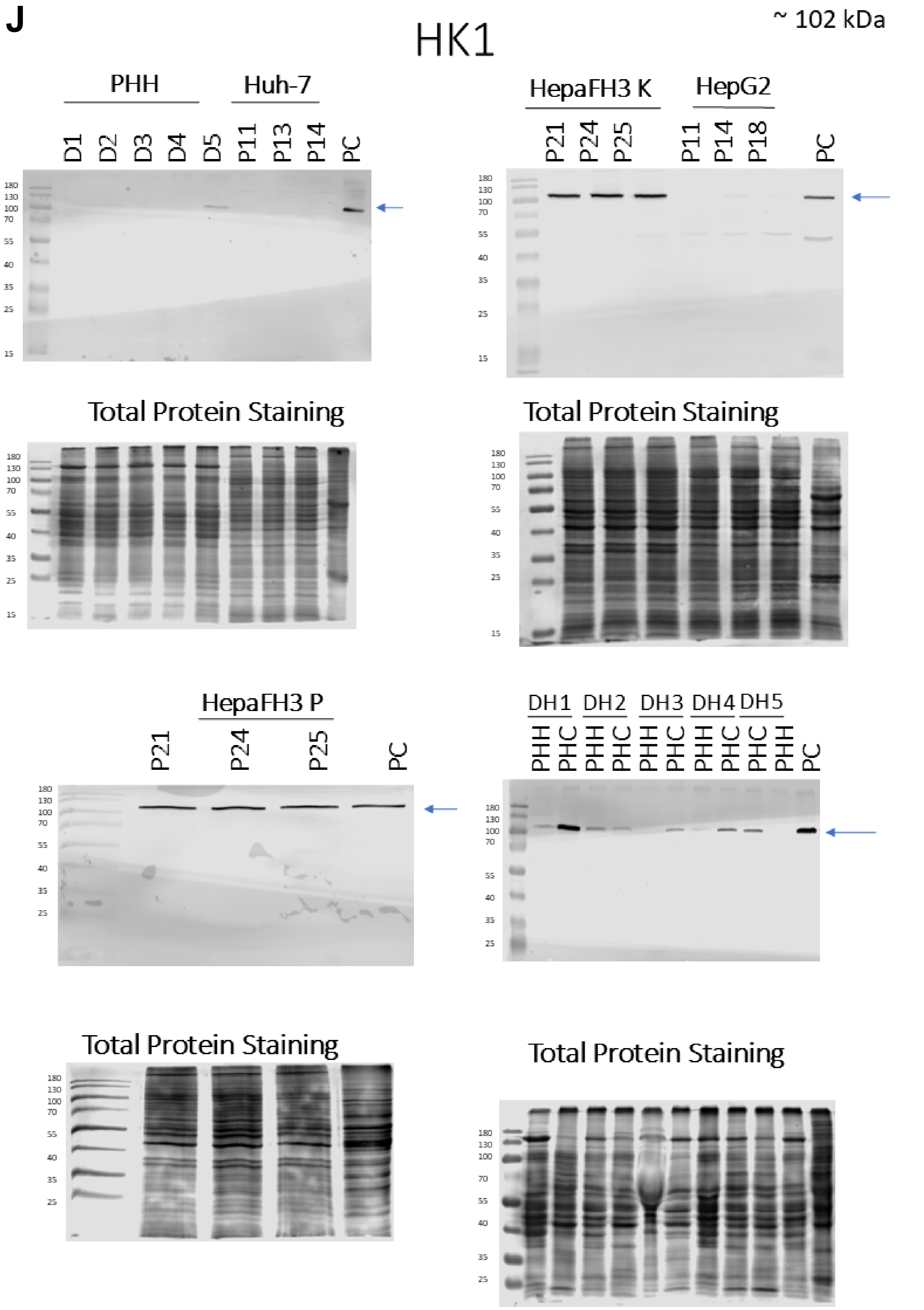
J
HK1
~ 102 kDa
PHH
Huh-7
D1 D2 D3 D4 D5 P11 P13 P14 PC
HepaFH3 K
HepG2
P21 P24 P25 P11 P14 P18 PC
180 130 100 70 55 40 35 25 15
Total Protein Staining
Total Protein Staining
HepaFH3 P
P21 P24 P25 PC
DH1 DH2 DH3 DH4 DH5
PHH PHC PHH PHC PHH PHC PHH PHC PHC PHH PC
Total Protein Staining
Total Protein Staining

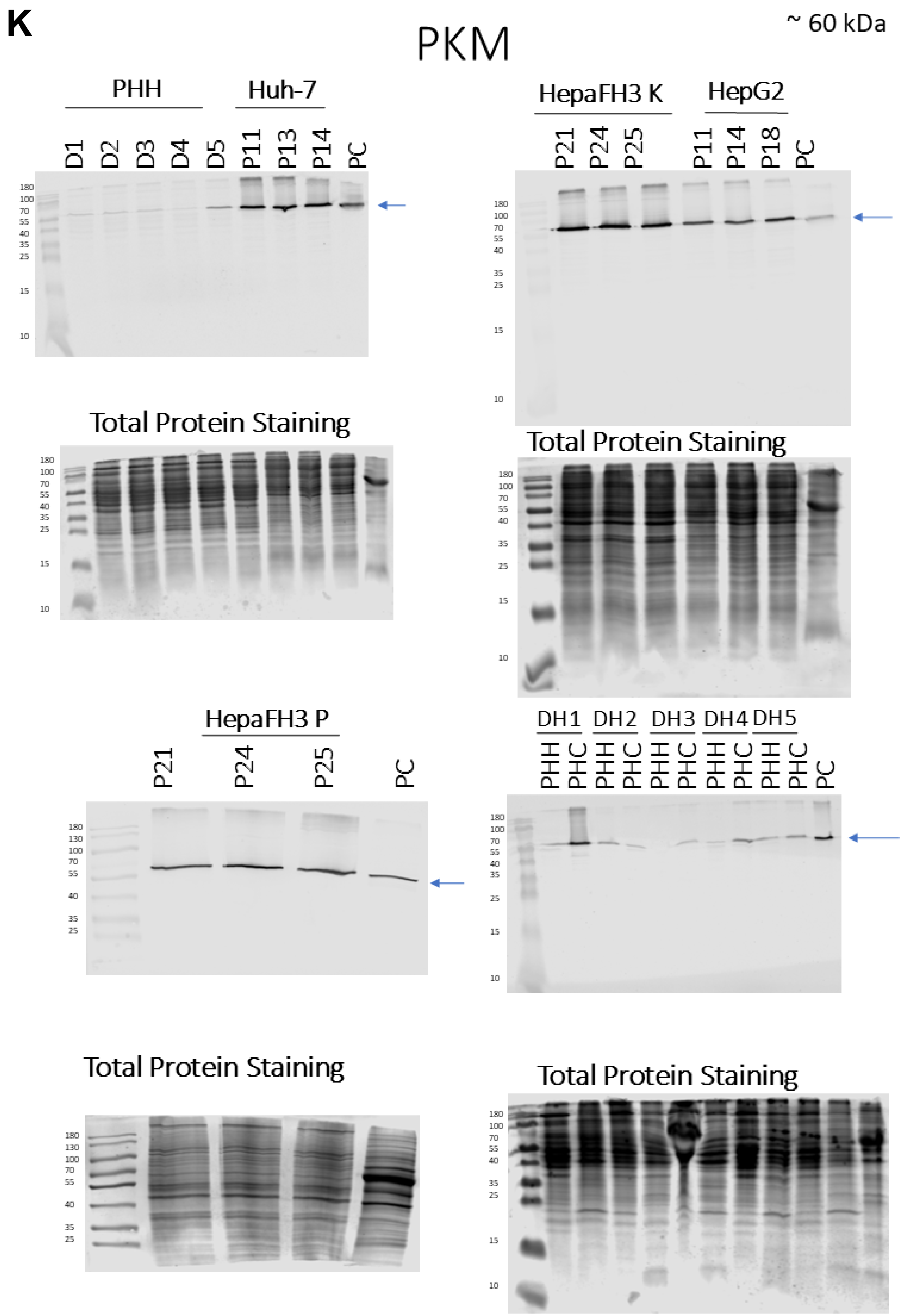
K
PKM
~ 60 kDa
PHH
Huh-7
D1 D2 D3 D4 D5 P11 P13 P14 PC
HepaFH3 K
HepG2
P21 P24 P25 P11 P14 P18 PC
Total Protein Staining
Total Protein Staining
HepaFH3 P
P21 P24 P25 PC
DH1 DH2 DH3 DH4 DH5
PHH PHC PHH PHC PHH PHC PHH PHC PHH PHC PC
Total Protein Staining
Total Protein Staining

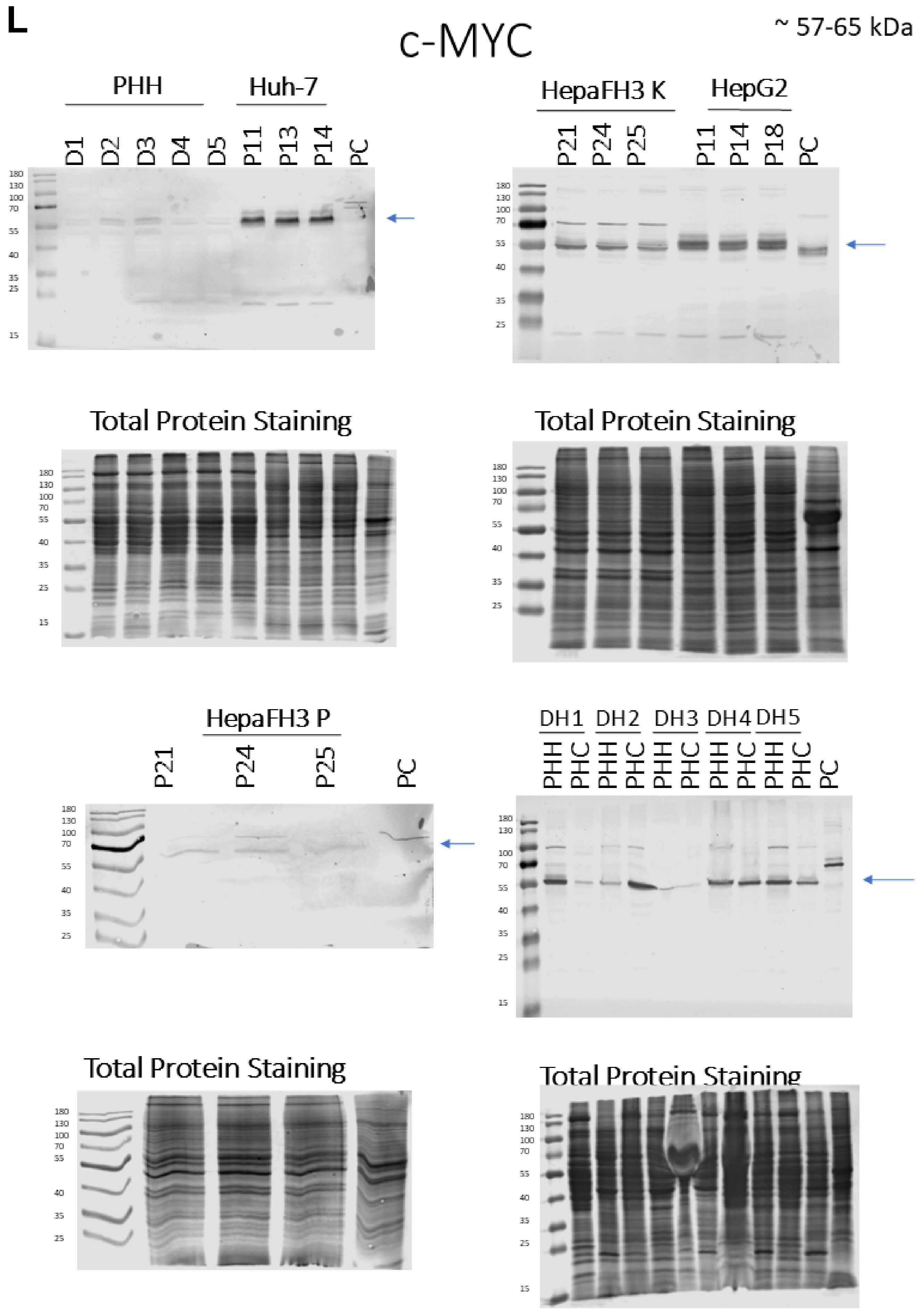
L
c-MYC
~ 57-65 kDa
PHH
Huh-7
D1 D2 D3 D4 D5 P11 P13 P14 PC
HepaFH3 K
HepG2
P21 P24 P25 P11 P14 P18 PC
Total Protein Staining
Total Protein Staining
HepaFH3 P
P21 P24 P25 PC
DH1 DH2 DH3 DH4 DH5
PHH PHC PHH PHC PHH PHC PHH PHC PHH PHC PC
180
130
100
70
55
40
35
25
15
Total Protein Staining
Total Protein Staining

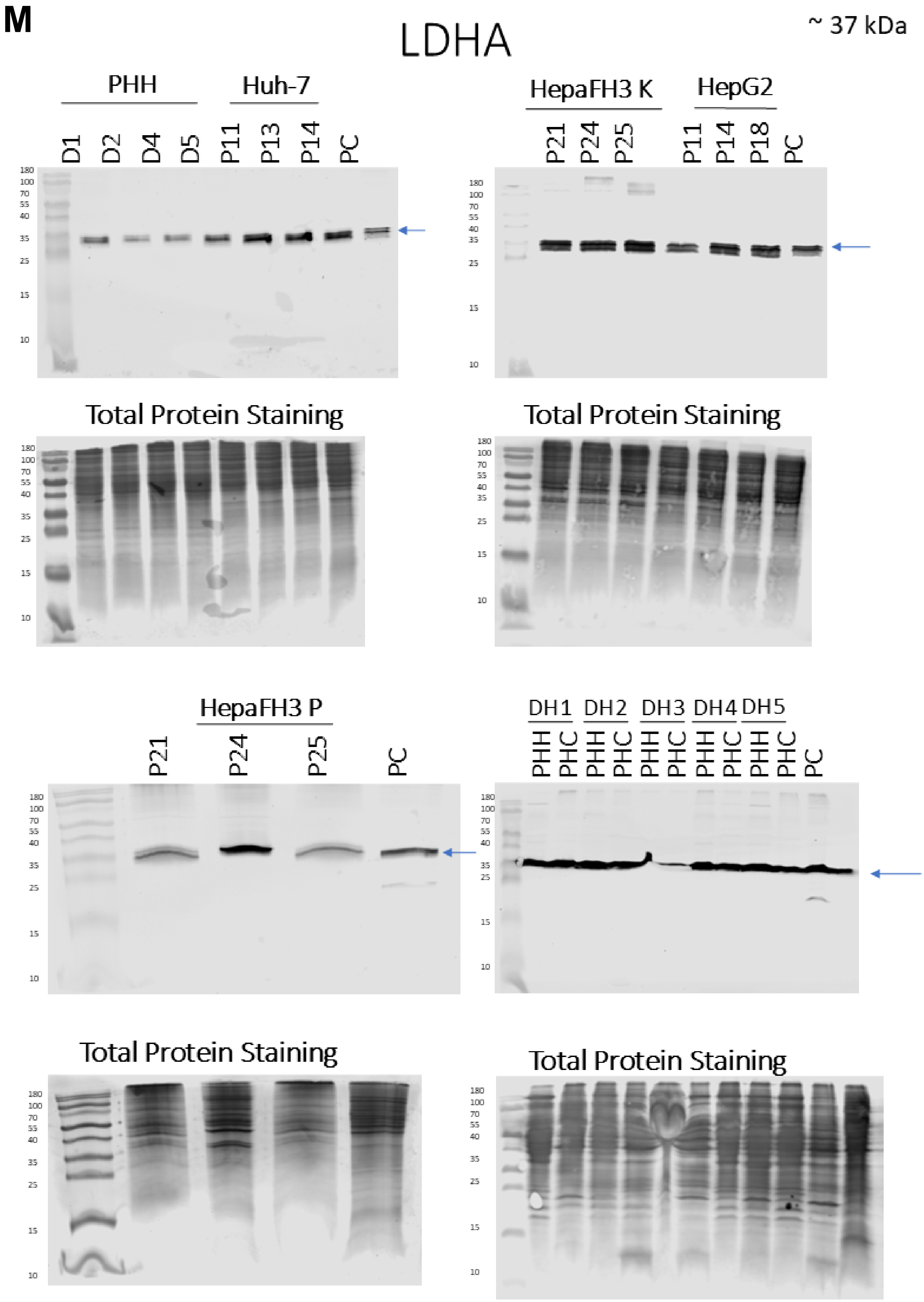
M
LDHA
~ 37 kDa
PHH
Huh-7
D1 D2 D4 D5 P11 P13 P14 PC
HepaFH3 K
HepG2
P21 P24 P25 P11 P14 P18 PC
180 100 70 55 40 35 25 15 10
Total Protein Staining
Total Protein Staining
HepaFH3 P
P21 P24 P25 PC
DH1 DH2 DH3 DH4 DH5
PHH PHC PHH PHC PHH PHC PHH PHC PHH PHC PC
Total Protein Staining
Total Protein Staining

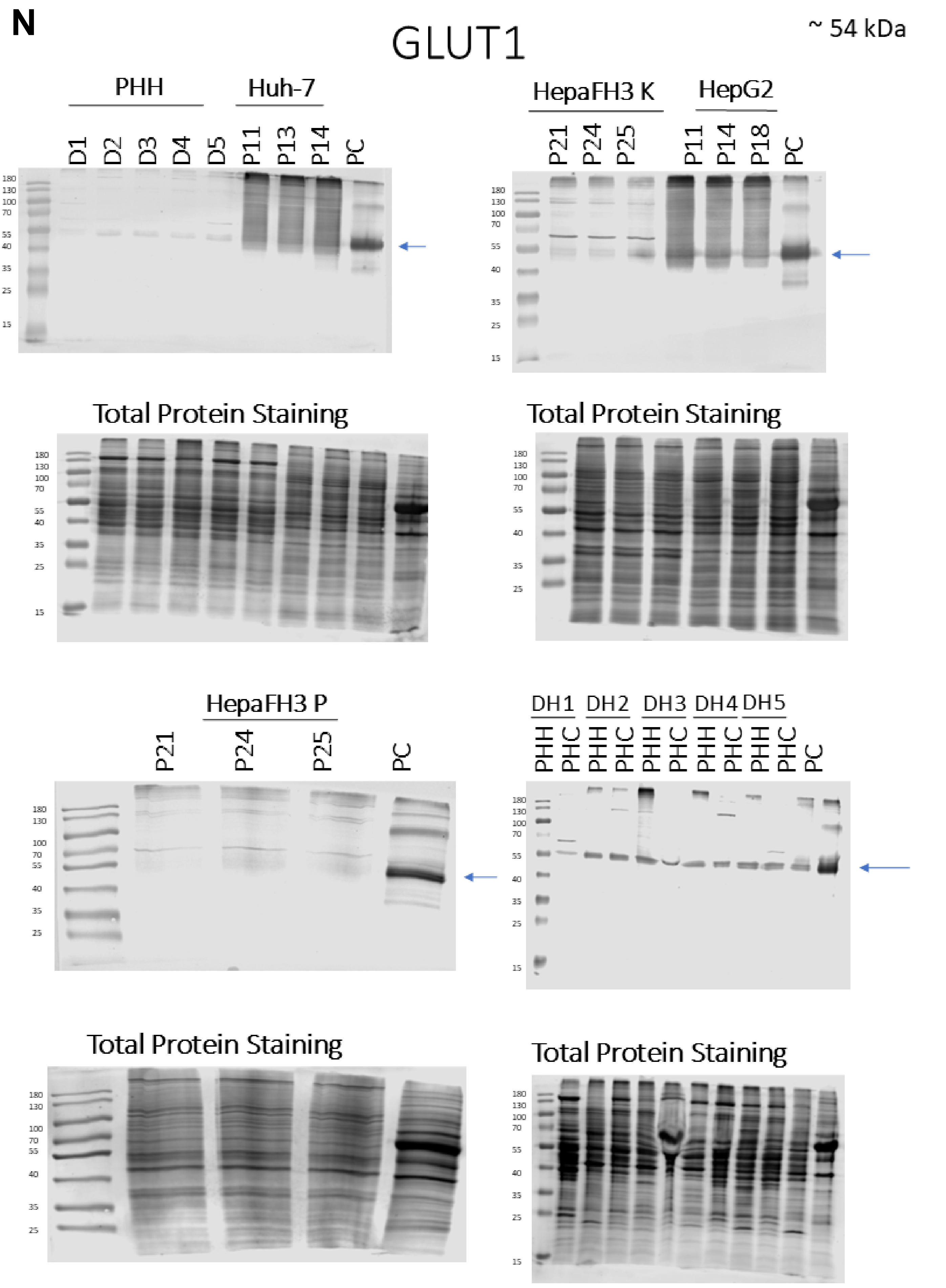
N
GLUT1
~ 54 kDa
PHH
Huh-7
D1 D2 D3 D4 D5 P11 P13 P14 PC
HepaFH3 K
HepG2
P21 P24 P25 P11 P14 P18 PC
Total Protein Staining
Total Protein Staining
HepaFH3 P
P21 P24 P25 PC
DH1 DH2 DH3 DH4 DH5
PHH PHC PHH PHC PHH PHC PHH PHC PHH PHC PC
Total Protein Staining
Total Protein Staining

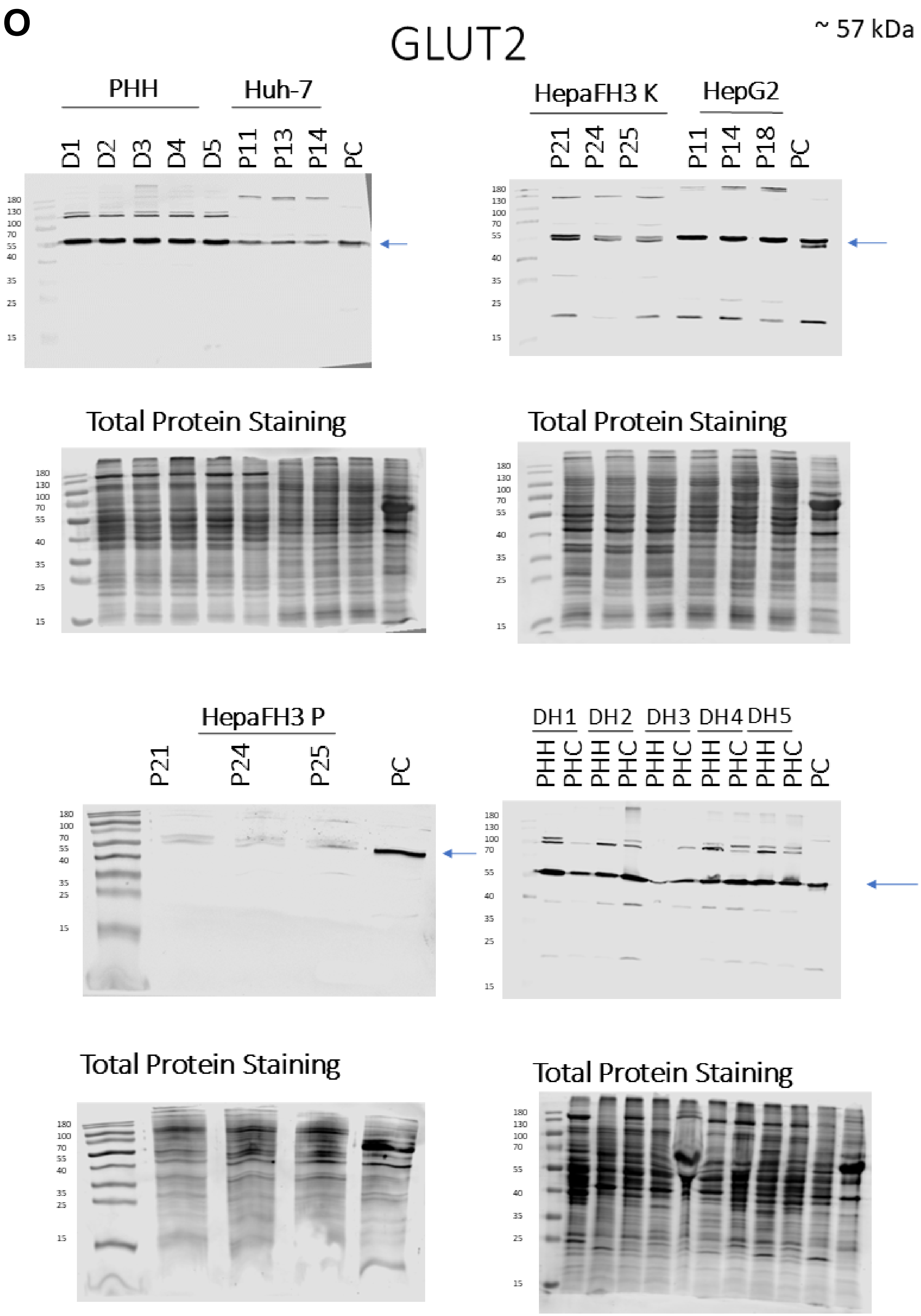
O
GLUT2
~ 57 kDa
PHH
Huh-7
D1 D2 D3 D4 D5 P11 P13 P14 PC
HepaFH3 K
HepG2
P21 P24 P25 P11 P14 P18 PC
180
130
100
70
55
40
35
25
15
Total Protein Staining
Total Protein Staining
HepaFH3 P
P21 P24 P25 PC
DH1 DH2 DH3 DH4 DH5
PHH PHC PHH PHC PHH PHC PHH PHC PHH PHC PC
Total Protein Staining
Total Protein Staining

**Figure S1.** Expression of various proteins in primary hepatic cells and hepatic cell lines. Western blot analyses were performed to assess the expression of (A) GSK3A, (B) HIF1A, (C) GSK3B, (D) HK2, (E) FOXO1, (F) GCK, (G) HMGCL, (H) BDH1, (I) PKL, (J) HK1, (K) PKM, (L) c-MYC, (M) LDHA, (N) GLUT1 and (O) GLUT2 in protein lysates from non-HCC primary human hepatocytes (non-HCC-PHHs, N = 5), HCC-derived PHHs (HCC-PHHs, N = 5), primary human hepatoma cells (PHCs, N = 5), Upcyte® hepatocytes HepaFH3 cells in confluent (HepaFH3 C, N = 3), and proliferating (HepaFH3 P, N = 3) states. Hepatoma cell lines HepG2 (N = 3) and Huh7 (N = 3 as well as a positive control (PC) were included for comparison. Arrows indicate the specific protein bands. Total protein staining is shown below each blot and was used for normalization. The positive control was also used for normalization purposes. Abbreviations: BDH1, 3-hydroxybutyrate dehydrogenase 1; C, confluent; c-MYC, MYC proto-oncogene; FOXO1, forkhead box O1; GCK, glucokinase; GLUT1, glucose transporter type 1; GLUT2, glucose transporter type 2; GSK3A, glycogen synthase kinase 3 alpha; GSK3B, glycogen synthase kinase 3 beta; HCC, hepatocellular carcinoma; HIF1A, hypoxia inducible factor 1 alpha; HK1, hexokinase 1; HK2, hexokinase 2; HMGCL, 3-hydroxymethyl-3-methylglutaryl-CoA lyase; LDHA, lactate dehydrogenase A; P, proliferating; PHC, primary human hepatoma cells; PHH, primary human hepatocytes; PKL, pyruvate kinase L; PKM, pyruvate kinase M1/2.

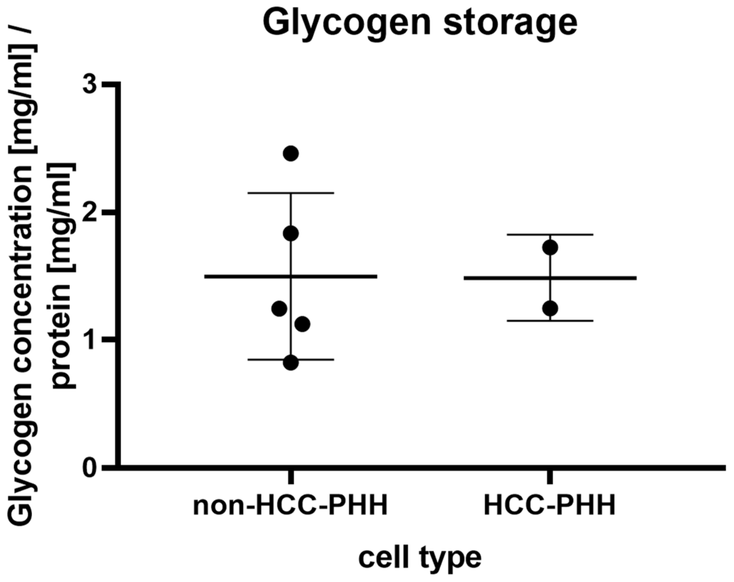


**Figure S2.** Glycogen levels in PHHs from non-HCC and HCC donors. Glycogen was quantified in cell lysates of non-HCC-PHHs (N = 5) and HCC-PHHs (N = 2). Values were normalized to total protein content. Abbreviations: HCC, hepatocellular carcinoma; PHH, primary human hepatocytes.

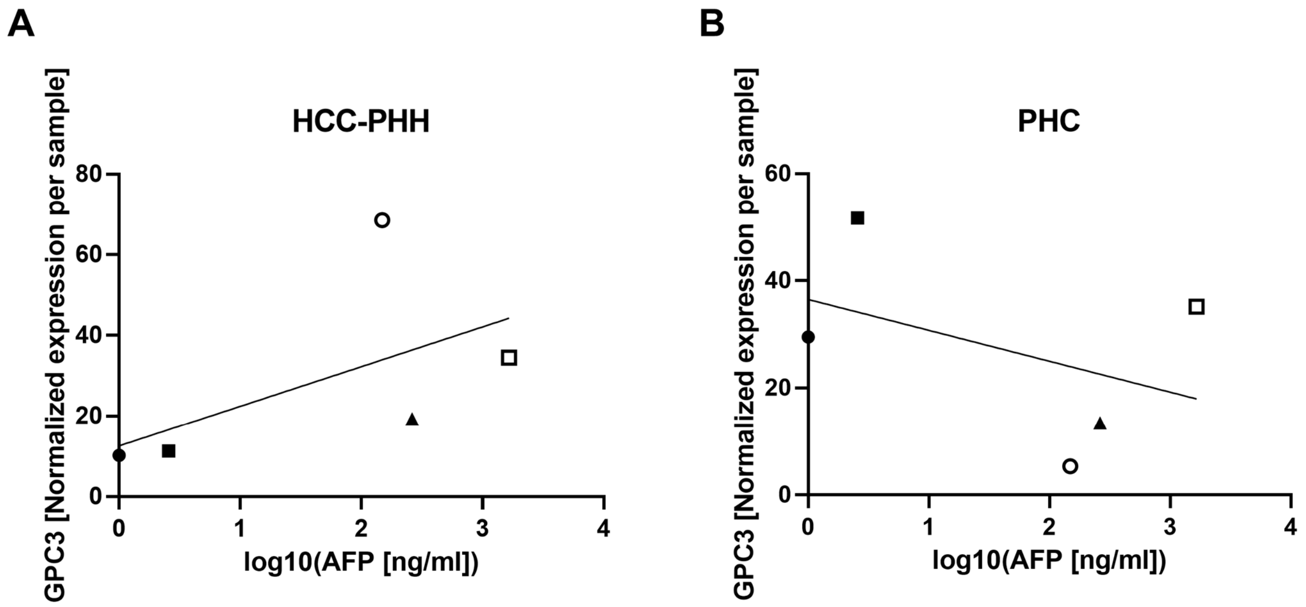


**Figure S3.** *GPC3* gene expression and AFP serum levels in HCC-PHHs and corresponding PHCs.

*GPC3* mRNA expression was quantified in HCC-PHHs (N = 4) and matched PHCs (N = 4) from individual donors. Preoperative serum AFP levels were measured in the same HCC donors. Pearson correlation analyses were performed between *GPC3* expression and AFP levels after log transformation of AFP values. (A) Correlation between *GPC3* expression in HCC-PHHs and log-transformed AFP levels (Pearson's r = 0.559, p = 0.3272). (B) Correlation between *GPC3* expression in PHCs and log-transformed AFP levels (Pearson's r = -0.4346, p = 0.4646). The data represent the individual donor values and are indicated of various symbols. Abbreviations: AFP, $\alpha$-fetoprotein; *GPC3*, glypican-3; HCC, hepatocellular carcinoma; PHCs, primary human hepatoma cells; PHHs, primary human hepatocytes.
